# Phosphoric Acid Doped Polybenzimidazole (PBI)/Zeolitic Imidazolate Framework Composite Membranes with Significantly Enhanced Proton Conductivity under Low Humidity Conditions

**DOI:** 10.3390/nano8100775

**Published:** 2018-09-29

**Authors:** Jorge Escorihuela, Óscar Sahuquillo, Abel García-Bernabé, Enrique Giménez, Vicente Compañ

**Affiliations:** 1 Escuela Técnica Superior de Ingenieros Industriales—Departamento de Termodinámica Aplicada, Universitat Politècnica de València, Camino de Vera s/n, 46020 Valencia, Spain; escorihu@uji.es (J.E.); agarciab@ter.upv.es (A.G.-B.); 2 Instituto de Tecnología de Materiales, Universitat Politècnica de València, Camino de Vera s/n, 46020 Valencia, Spain; ossana@upvnet.upv.es (Ó.S.); enrique.gimenez@mcm.upv.es (E.G.)

**Keywords:** proton exchange membrane, polybenzimidazole, zeolitic imidazolate framework, proton conductivity

## Abstract

The preparation and characterization of composite polybenzimidazole (PBI) membranes containing zeolitic imidazolate framework 8 (ZIF-8) and zeolitic imidazolate framework 67 (ZIF-67) is reported. The phosphoric acid doped composite membranes display proton conductivity values that increase with increasing temperatures, maintaining their conductivity under anhydrous conditions. The addition of ZIF to the polymeric matrix enhances proton transport relative to the values observed for PBI and ZIFs alone. For example, the proton conductivity of PBI@ZIF-8 reaches 3.1 × 10^−3^ S·cm^−1^ at 200 °C and higher values were obtained for PBI@ZIF-67 membranes, with proton conductivities up to 4.1 × 10^−2^ S·cm^−1^. Interestingly, a composite membrane containing a 5 wt.% binary mixture of ZIF-8 and ZIF-67 yielded a proton conductivity of 9.2 × 10^−2^ S·cm^−1^, showing a synergistic effect on the proton conductivity.

## 1. Introduction

The study of proton conductivity processes has gained intense attention in the past decades due to their potential application in chemical sensors, electrochemical devices and energy generation and storage [1]. Principally, proton conducting membranes have attracted increasing interest in different areas such as fuel cells, batteries and supercapacitors [2]. Among the different types of fuel cells, proton exchange membrane fuel cell (PEMFC) are of utmost interest due to their use in transportation and stationary power generation applications [3]. In a typical PEMFC, the polymeric electrolyte membrane is responsible for the proton conductivity, which allows the transport from anode to cathode, and it consequently constitutes the essential component of the electrochemical device [4,5]. Among the different varieties of electrolyte membranes, those based on perfluorosulfonic acid (PFSA) polymers, such as Nafion^®^, have been widely used in low temperature proton exchange membrane fuel cells (LT-PEMFCs), due to their high conductivity and good chemical and mechanical properties at moderate temperatures below 90 °C and high relative humidity conditions [6]. The main drawbacks of Nafion^®^ membranes are their high cost, hazardous manufacturing processes as well as their decrease in proton conductivity at temperatures above 90 °C under low humidity conditions [7,8]. As a consequence, high temperature proton exchange membrane fuel cells (HT-PEMFCs), which are able to operate in the range of 160–200 °C without humidity, have become the focus of intense research efforts [9,10]. The benefits of working at high temperatures include the reduction in catalyst poisoning, faster electrode kinetics and ease associated with water and thermal management [11,12]. In this regard, various types of HT-PEMFC membranes have been developed to overcome all of these problems, and particularly those based on polybenzimidazole (PBI) have emerged as promising candidates [13,14].

PBI is a synthetic polymer with aromatic and heteroaromatic rings with a very high glass transition temperature (425–436 °C) and excellent chemical and thermal stability. PBI membranes exhibit low proton conductivities under low humidity conditions. However, they can be significantly increased when doped with phosphoric acid (PA) [15,16,17]. The excellent stability of acid-doped PBI combined with its high proton conductivity has promoted their study as a superior alternative to HT-PEMFC membranes (especially from 150 to 200 °C) among other polymeric electrolytes, such as poly(arylene ether) and polyimide-based membranes [18,19]. Even so, one alternative to improve proton PBI conductivity is based on developing mixed-matrix membranes (MMMs), which are composite membranes made by combining an inorganic–organic hybrid material and a polymer matrix [20]. In particular, PBI-based MMMs have been recently used for various applications such as nanofiltration [21], pervaporation [22], organocatalysis [23] and fuel cells [24]. In recent years, the use of metal organic frameworks (MOFs) as fillers in polymeric electrolyte membranes has attracted a growing interest due to their high conductivity, which is mainly attributed to their high porosity [25,26,27].

**Figure 1:**
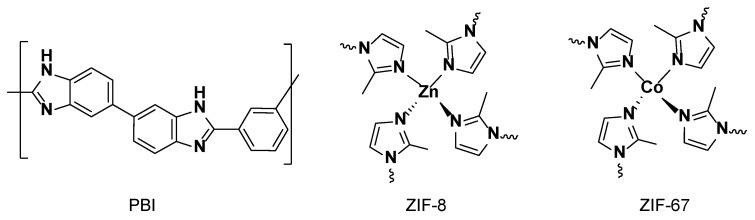
Chemical structure of the polybenzimidazole (PBI) polymer repeating unit, the zeolitic imidazolate framework 8 (ZIF-8), and zeolitic imidazolate framework 67 (ZIF-67).

MOFs are a family of crystalline materials based on the combination of metal-containing units with organic linkers capable of forming strong bonds and leading to the creation of open crystalline frameworks with regular porous structures [28]. The understanding and study of MOFs have experienced an exponential growth in the past decade, particularly due to their use in many applications, such as catalysis, separation technology, gas storage, drug delivery, etc. [29,30]. One subclass of MOFs is zeolitic imidazolate frameworks (ZIFs), which represent a unique class of materials with a zeolitic topology combining a tetrahedral divalent metal cation M^2+^ (M = Co and Zn) coordinated to four imidazolate rings to form neutral porous framework structures (M(Im)_2_) with high chemical and thermal stability (Figure 1) [31,32]. The high microporosity of the cavities of the ZIFs is a factor of interest for the inclusion of guest molecules to favor proton conduction through the framework structure. In this regard, the incorporation of ZIFs into polymeric composite membranes is considered a significant step toward their potential use in fuel cell applications, as well as in liquid and gas separation due to the increased porosity as a result of the MOF incorporation. To date, only a few MOF−polymer composite membranes based on perfluorosulfonic acid (PFSA) polymers [33,34,35,36], sulfonated poly(ether ether ketone) (SPEEK) [37,38,39,40,41,42], poly(vinyl alcohol) (PVA) [43,44,45], and other polymeric materials have been reported to achieve PEMFCs [46,47,48]. Among the reported MOF-containing PEMs, proton conductivities as high as 0.3 S·cm^−1^ have been reported for Nafion^®^ and SPEEK membranes under 100% relative humidity (RH) and temperatures below 80 °C (Tables S1–S3, Supporting Information) [33,34,37]. However, under anhydrous conditions only values of 3 × 10^−3^ S·cm^−1^ have been reached for MOF composite membranes (Figures S4–S6, Supporting Information).

Herein, we report on the preparation and characterization of the proton conductivity of composite PBI-based membranes in which the MOF particles are embedded in the polymeric matrix. We selected zeolitic imidazolate framework 8 (ZIF-8) and zeolitic imidazolate framework 67 (ZIF-67), as they have been widely recognized for their gas separation performances. For that, a Zn-based ZIF-8, a Co-based ZIF-67, and a ZIF-mix, a Zn/Co bimetallic zeolitic imidazolate framework, were synthesized for the preparation of novel PBI composite membranes, which were doped with phosphoric acid and tested as proton conducting membranes suitable for applications at high temperatures under anhydrous conditions. To the best of our knowledge, the reported conductivities in this work are among the highest reported for MMMs in HT-PEMFCs.

## 2. Materials and Methods 

### 2.1. Materials 

Zinc nitrate tetrahydrate (for an EMSURE^®^ analysis), 2-Methylimidazole (Hmim with 99% purity), cobalt nitrate hexahydrate (99.999% trace metals basis), and lithium chloride (LiCl) were purchased from Sigma-Aldrich (Sigma-Aldrich Química SL, Madrid, Spain). *N*,*N*-Dimethylacetamide (DMAc, 99.5% extra pure) and phosphoric acid (extra pure, 85% solution in water) were purchased from Acros Organics (Fisher Scientific SL, Madrid, Spain). PBI (purity > 99.95%, MW 65000, with the molecular formula: (C_20_H_12_N_4_)_n_) was purchased from Danish Power Systems (Danish Power Systems, Kvistgaard, Denmark).

### 2.2. Characterization

Powder X-ray diffraction (XRD) was acquired using a D/Max-2500PC diffractometer (Rigaku Europe SE, Neu-Isenburg, Germany) with Cu Kα radiation (λ = 1.5406 Å) in the 2θ range between 10° and 70°, with a scanning rate and step size of 2° min^−1^ and 0.02°, respectively. Scanning electron microscopy (SEM) images were acquired on an FE-SEM model Ultra 55 (Zeiss, Oberkochen, Germany) field emission scanning electron microscope FE-SEM model Ultra 55 (Zeiss, Oberkochen, Germany) operating at 5 kV with energy-dispersive X-ray (EDX) spectroscopy. Electron micrographs were obtained using a Jeol JEM-1010 high resolution microscope (JEOL Ltd., Garden City, UK). Attenuated total reflection Fourier transform infrared (ATR-FTIR) spectra of the membranes were recorded on a Jasco FT-IR spectrometer FT/IR-6200 Series (Jasco Spain, Madrid, Spain) with a 4 cm^−1^ resolution between 400 and 4000 cm^−1^. Thermogravimetric analysis (TGA) was performed on a TGA Q50 thermogravimetric analyzer TGA Q50 (Waters Cromatografia, S.A., Division TA Instruments, Cerdanyola del Valles, Spain). The samples (5–10 mg) were weighed in zirconia crucibles and were heated under nitrogen atmosphere from 25 to 800 °C at a heating rate of 10 °C·min^−1^. For the surface area and porosity analysis, the solid or membrane was dried in a vacuum oven at 100 °C for 5 h and activated at 100 °C for 12 h on a SmartVacPrep instrument (Micromeritics Instrument Corporation, Norcross, GA, USA). All of the N_2_ isotherms were measured on a Micromeritics Tristar II (Micromeritics, Norcross, GA, USA) at room temperature. The consistency criteria were adapted to choose the pressure range selection for a Brunauer–Emmett–Teller (BET) calculation. The acid uptake (AU) of the membrane was calculated by the following equation: AU (%) = [(W_wet_ − W_dry_)/W_dry_] × 100; where W_wet_ and W_dry_ refer to the membrane’s weight after its immersion in phosphoric acid for at least 48 h at room temperature and the membrane’s weight after drying at 120 °C for at least 24 h, respectively. Acid leaching tests for all of the membranes were carried out in order to determine the phosphoric acid retention capability of the prepared composite membranes. This is considered one of the main degradation factors of PBI membranes in the HT-PEMFC. For that purpose, acid leaching experiments were conducted by weighting the phosphoric acid doped membranes after their immersion in boiling water for a period of 5 h. The weight of the acid leached membranes was noted every hour. The oxidative stability of the membranes was investigated by immersing the membranes in Fenton’s reagent (3% H_2_O_2_ solution containing 4 ppm Fe^2+^) at 70 °C. The samples were collected by filtering and rinsed with deionized water several times, then dried at 120 °C for 5 h in a vacuum oven. Next, the degradation of the membranes was evaluated by their weight loss. The tensile tests from each thin-film composite membrane were performed using a Shimadzu AGS-X Universal Testing Machine (Izasa Scientific, Madrid, Spain). The mechanical parameters were determined from the average of five samples. For all of the tests, a tensile speed of 5 mm/min and a load cell of 500 N were used. The proton conductivity measurements of the membranes in the transversal direction were performed in the temperature range between 0 and 200 °C by electrochemical impedance spectroscopy (EIS) in the frequency interval of 10^−1^ < f < 10^7^ Hz, applying a 0.1 V signal amplitude. A broadband dielectric spectrometer (Novocontrol Technologies, Hundsangen, Germany) integrated with an SR 830 lock-in amplifier with an Alpha dielectric interface was used. The membranes were previously immersed in deionized water and the thickness was measured afterwards using a digital micrometer, taking the average measurements at different parts of the surface. Then, the membranes were placed between two gold electrodes coupled to the spectrometer. Initially, the temperature was gradually raised from 20 °C to 120 °C in steps of 10 °C and the dielectric spectra were collected at each step. During the second cycle of temperature scan (named as anhydrous conditions in the manuscript), the dielectric spectra were collected at each step. second cycle of the temperature scan (called “anhydrous conditions” in the manuscript), the dielectric spectra were collected at each step from 0 °C to 200 °C, in steps of 10 °C.

### 2.3. Experimental Procedures

#### 2.3.1. Synthesis of the ZIF-8

A solution of 2-methylimidazole (4.25 g, 51.5 mmol) was dissolved in an ethanol:methanol mixture (*v*/*v* 1:1, 50 mL) and then was then poured into a solution of Zn(NO_3_)_2_·6H_2_O (2.10 g, 7.1 mmol) in 50 mL of a binary ethanol:methanol mixture. The resulting solution was vigorously stirred at room temperature for 4 h. At the end of the stirring process, the solution was centrifuged at 4500 rpm (30 min) and washed with ethanol and hexane several times, in order to obtain the desired white powder (Figure S1, Supporting Information). Finally, the white solid was vacuum-dried in a vacuum oven at 90 °C for 24 h. ATR-FTIR: 3465, 3130, 2935, 2859, 2765, 2366, 2325, 1693, 1591, 1458, 1421, 1392, 1311, 1181, 1146, 1031, 995, 956, 766, 694, 685, 613 and 423 cm^−1^.

#### 2.3.2. Synthesis of the ZIF-67 

A solution of 2-methylimidazole (2.07 g, 25.2 mmol) was dissolved in an deionized water and was then poured into a 50 mL deionized water solution of Co(NO_3_)_2_·6H_2_O (1.51 g, 6.3 mmol). The resulting solution was vigorously stirred at room temperature for 1 h and maintained at room temperature for 24 h. The solution was centrifuged at 4500 rpm (30 min) and the resulting purple precipitates were collected and washed with ethanol and hexane several times. Finally, the solid was vacuum-dried at 90 °C for 24 h (Figure S1, Supporting Information). ATR-FTIR: 3374, 3130, 2925, 1678, 1590, 1508, 1455, 1419, 1380, 1303, 1176, 1140, 1126, 1096, 1011, 990, 754, 682, 647, 622 and 427 cm^−1^.

#### 2.3.3. Preparation of the PBI Solution 

LiCl (0.1 wt.%) as a stabilizer was dissolved in DMAc with vigorous stirring to give a homogeneous solution. Next, PBI powder (10 wt.%) was dissolved in the LiCl solution (in DMAc) and heated under reflux at 120 °C for 6 h. The prepared solution had a viscosity of 0.5 Pa·s at 25 °C.

#### 2.3.4. Membrane Preparation 

The corresponding amount (wt.%) of zeolitic imidazolate framework (ZIF-8, ZIF-67, or ZIF-mix) was dissolved in the PBI solution and placed in an ultrasonic bath for 1 h and next, the mixture was then stirred for 3 h at 60 °C. Then, the solution was cast onto a glass slide and dried at 70 °C for 10 h, then at 140 °C for 10 h, and finally at 120 °C under vacuum overnight (Figure S2, Supporting Information).

## 3. Results and Discussion

### 3.1. Characteization of the Membranes

ZIF-8 and ZIF-67 are isostructural MOFs (i.e., crystallographically the same structure) with different metal nodes (Zn^2+^ in ZIF-8 and Co^2+^ in ZIF-67). Powder X-ray diffraction (PXRD) was used to characterize the synthesized ZIFs and PBI composite membranes. The powder diffraction peaks obtained for the synthesized ZIF-8 and ZIF-67 materials are in accordance with the XRD pattern reported for these two imidazolate derivatives (Figure S3, Supporting Information) and confirm the expected solid structure of both of the zeolitic imidazolate frameworks [49,50]. The PXRD of the mixed matrix membranes showed the incorporation of ZIF into the polymeric matrix.

**Figure 2:**
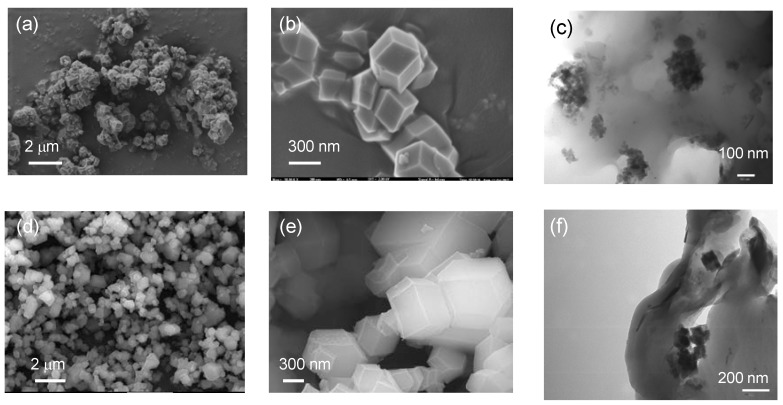
(**a**) Low and (**b**) high-magnification field-emission scanning electron microscopy (FE-SEM) images of the ZIF-8; (**c**) transmission electron microscopy (TEM) image of the PBI composite membrane containing ZIF-8 (5 wt.%); (**d**) low and (**e**) high-magnified FE-SEM images of the ZIF-67; (**f**) TEM image of the PBI composite membrane containing ZIF-67 (5 wt.%).

The size and morphology of the synthesized ZIF-8 and ZIF-67 particles was investigated by field-emission scanning electron microscopy (FE-SEM) and transmission electron microscopy (TEM). Figure 2 shows the FE-SEM micrographs of the ZIF powders, in which we can see the typical ZIF polyhedrons with a defined rhombic dodecahedral shape. The average particle size obtained by SEM for the ZIF-8 and the ZIF-67 was around 350–400 nm. Particle size is a critical factor in the properties and stability of the colloidal solution because smaller particles favor a better dispersion in the solution, and finally in the polymeric matrix upon the evaporation of the solvent. Additionally it is possible to achieve a larger effective contact area for transport carriers by using smaller particles. The incorporation of synthesized ZIF particles into the PBI matrix showed that they have a certain tendency to form small aggregates, as revealed by TEM (Figure 2c,f). Elemental analysis and EDX of the composite membranes revealed the presence of metallic atoms (Zn and Co) in the polymeric matrix (Figures S4–S6, Supporting Information).

ATR/FT-IR was also used to characterize the composite membranes. In the IR spectrum of the pure PBI membrane, a broad peak around 3500–3200 cm^−1^ (N–H stretching) and bands at 1607 and 1421 cm^−1^ (C=N and C–N stretching, respectively) were observed. Additionally, the absorption band corresponding to the stretching modes of the imidazolate ring (C–C) was also visible at 1453 cm^−1^ [51]. The metallic bands for the ZIF-8 (Zn–C) and the ZIF-67 (Co–C) could be observed around 425 cm^−1^, confirming the presence of the ZIF in the polymeric matrix (Figures S7–S11, Supporting Information). The porosity of the PBI@ZIF-8, PBI@ZIF-67 and PBI@ZIF-mix compared to the synthesized ZIF-8, ZIF-67 and ZIF-mix was determined by N_2_ isotherm measurements (Table S4, Supporting Information). The measured specific surface areas (BET areas) for ZIF-8, ZIF-67 and ZIF-mix were 1151, 1379 and 1171 m^2^·g^−1^, respectively. In contrast, for the PBI membranes specific surface areas of the PBI membranes decreased dramatically, which implies that the ZIF pores were blocked by the PBI polymer in the membranes.

**Figure 3:**
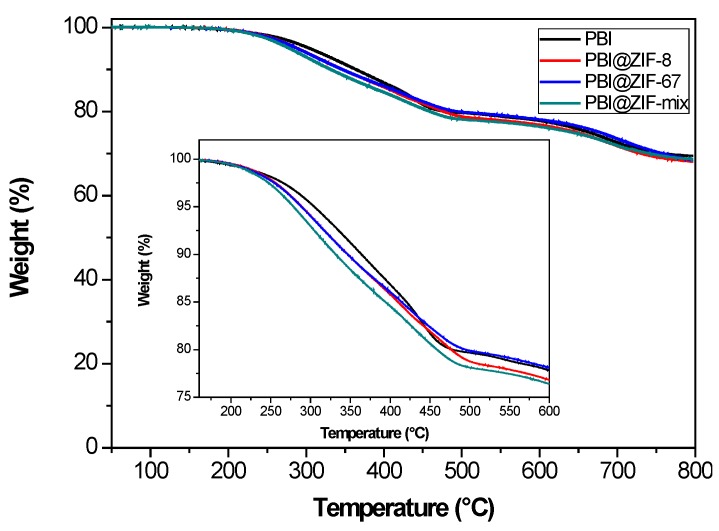
Thermogravimetric analysis of PBI membrane and PBI composite membranes containing 5 wt.% of ZIF-8, ZIF-67 and ZIF-mix. Inset: Zoom at the 180–600 °C region.

According to the thermogravimetric analysis (TGA) under a N_2_ atmosphere, only one degradation step was observed around 550–600 °C for the pristine PBI. This was attributed to the decomposition of the main polymer chain (Figure S12, Supporting Information). However, the pure PBI membrane showed a two-step degradation pattern (Figure 3). A first decomposition stage with a weight loss of 20% was found between 250 and 470 °C, with a 2% loss in the range of temperature from 50 to 250 °C, basically due to the dehydration of the absorbed water molecules and DMAc traces [52]. Finally, a degradation step occurred around 610 °C with a 70% of the weight remaining weight at 800 °C. This can be attributed to the degradation of the PBI main chain [53]. As shown, no alteration in the degradation patterns of the PBI was observed upon the addition of the ZIF (5 wt.%) to the PBI matrix. Higher losses were observed in the 450–600 °C range, attributed to the thermal decomposition of the ZIF compounds (Figures S13–S15, Supporting Information). After several decomposition stages, the membranes retained 65% of their weight at 800 °C. In summary, the TGA studies reveal that these composite membranes have enough thermal stability and that they are suitable for the operation at high temperatures (120–180 °C).

The stress vs. strain graph of the PBI and PBI@ZIF composite membranes containing 5 wt.% of ZIF-8, ZIF-67, and ZIF-mix is shown in Figure S16 (Supporting Information). Note that the mechanical properties were studied prior to the membrane doping with phosphoric acid under dry conditions (samples were dried overnight in an oven at 120 °C). The corresponding Young’s modulus, tensile strength, elongation at break, and toughness values are presented in Table 1. Compared to the neat PBI membrane under dry conditions, the Young’s modulus and tensile strength values of the PBI@ZIF composite membranes decreased. The tensile strength of the PBI@ZIF-8 and PBI@ZIF-67 decreased by around 53% and 56%, respectively, as compared to neat dry PBI. However, a slightly lower decrease (50%) was observed in the PBI@ZIF-mix. This behavior changes when compared to the neat PBI with a certain relative humidity, showing a higher elastic modulus for the PBI@ZIF composite membranes. The presence of water in the ZIFs might have affected the interfacial interactions between the ZIFs and the PBI. This fact may suggest a plasticization effect on the PBI@ZIF membranes, as shown by the decrease in the Young’s modulus and tensile strength, together with a significant increase in the elongation at break compared to the neat PBI in dry conditions. The improvement in the toughness of the PBI@ZIF-mix membrane as compared to the dry PBI can be attributed to the good interfacial interaction between both types of ZIF particles and the PBI matrix, and may indicate the presence of water molecules adsorbed in the ZIF cavities. Regarding the humid PBI (75% RH), the PBI@ZIF-mix membrane also showed a slightly improved toughness, which may be beneficial for the application of the material in fuel cells in humid operating conditions.

**Figure 4:**
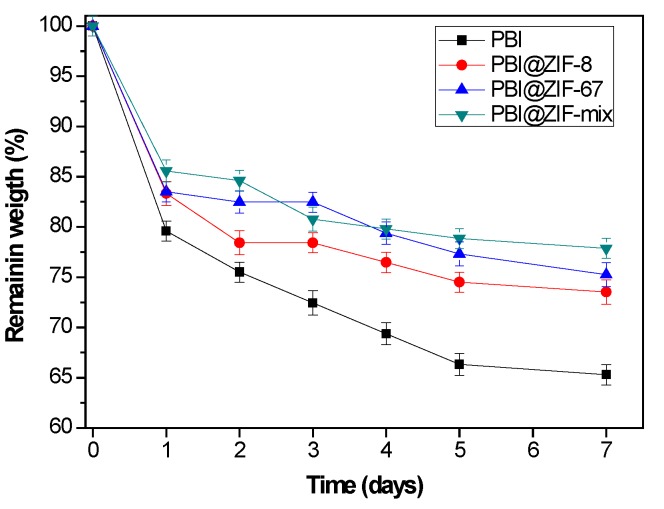
Fenton’s test (3% H_2_O_2_ containing 4 ppm Fe^2+^ at 70 °C) for membrane degradation.

Oxidative stability is considered a critical parameter in the fabrication of novel HT-PEMFCs as it affects the long-term operation and consequently the lifetime and the performance of membranes. In this regard, Fenton’s test was used to investigate the chemical stability of the polymeric membranes in order to evaluate their long-term durability. Therefore, the oxidative stability of the membranes was investigated by immersing the samples in Fenton’s reagent (3% H_2_O_2_ solution containing 4 ppm Fe^2+^) at a temperature of 70 °C and the oxidative stability of the membranes was calculated by their weight loss. As shown in Figure 4, all of the ZIF-containing membranes displayed a similar level of oxidative stability, but it was better than that observed for the pristine PBI membrane. Among the three mixed-matrix membranes containing ZIFs, the PBI@ZIF-mix displayed the best behavior.

### 3.2. Phosphoric Acid Doping of the Membranes

**Figure 5:**
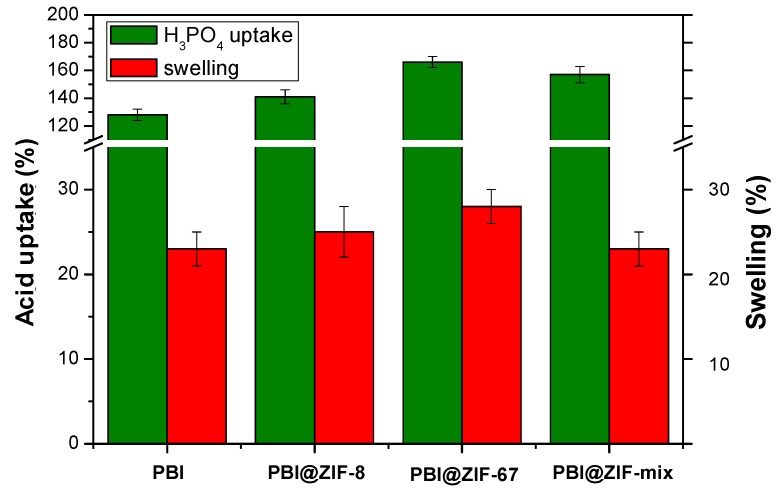
The acid uptake and swelling ratios of the PBI and PBI composite membranes containing 5 wt.% of ZIFs after their immersion in a phosphoric acid solution (85%) at room temperature after 48 h.

As mentioned above, the proton conductivity of PBI membranes can be increased by their immersion in phosphoric acid. Therefore, the PBI composite membranes were immersed in a concentrated aqueous phosphoric acid solution (85%) at room temperature. After 48 h, the acid uptake remained constant, indicating that the equilibrium had been reached. For the pure PBI membrane, the acid uptake was 128% with a 23% swelling ratio. In order to study the effect of the incorporation of ZIF, membranes containing ZIF-67 at different proportions (0.5, 1, 5 and 10 wt.%) were prepared and immersed in phosphoric acid (Figure S17, Supporting Information). Results showed that the acid uptake increased when the content of ZIF-67 in the PBI membrane increased, whereas the swelling ratio remained similar for all ZIF-67 contents (26–28%). When comparing PBI composite membranes with 5 wt.% to the three different the ZIFs (Figure 5), PBI@ZIF-67 membrane exhibited a higher acid uptake (166%) than the PBI@ZIF-8 (141%) and the PBI@ZIF-mix (157%), suggesting a higher affinity of phosphonate anion to bind to Co (II) rather than to Zn (II).

For the PA-doped membranes, all of the decomposition curves display a similar trend. The first degradation step was observed at 160 °C. It was attributed to the condensation reaction of phosphoric acid to form pyrophosphoric acid. The second step observed at about 600 °C was associated with the degradation of the PBI main chain and the continuous dehydration of the pyrophosphone acid to polyphosphoric acid (Figure S18, Supporting Information).

It is well known that phosphoric acid is a good proton carrier, especially when operating at elevated temperatures (Figure S19, Supporting Information). However, the long-term operation of the PA-doped membranes could be affected by the progressive release of the PA. Acid leaching tests for all the membranes were carried out for all of the membranes in order to determine the phosphoric acid retention capability of the prepared composite membranes. The phosphoric acid retention capability of PBI membranes is considered one of the main degradation factors in HT-PEMFCs. For that purpose, acid leaching experiments were conducted by weighting the phosphoric acid-doped membranes after their immersion in boiling water. The PBI@ZIF hybrid membrane retained higher amounts of the acid than the pristine PBI membrane (Table S5, Supporting Information). The degree of acid leaching of the PBI membrane was found to be around 40%. In contrast, for the PBI@ZIF composite membranes, the degree of acid leaching was reduced, suggesting that ZIF compounds could trap more acid molecules and prevent them from leaching out of the membrane, as observed with other PBI hybrid membranes with fillers such as SiO_2_ [54,55].

### 3.3. Proton Conductivity

**Figure 6:**
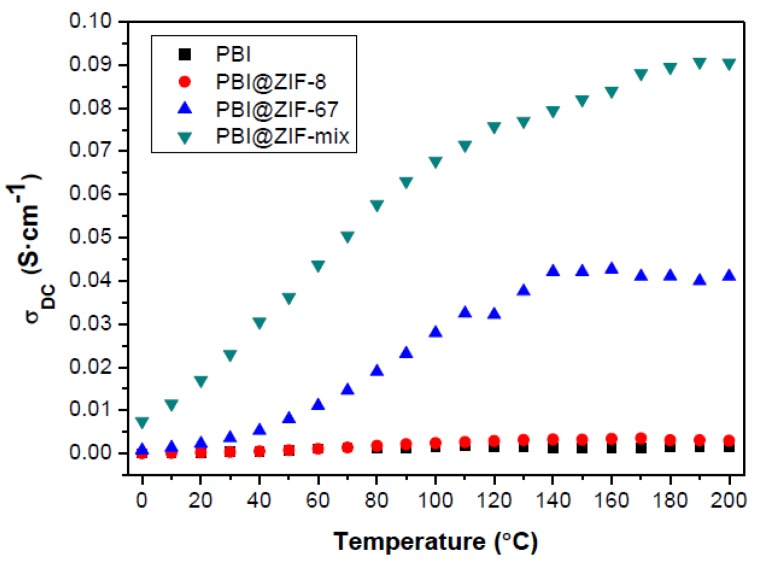
The temperature dependence of the proton conductivity of phosphoric acid doped PBI composite membranes containing 5 wt.% of ZIFs.

Proton conductivity is an important feature to study in order to evaluate the performance of novel proton exchange membranes (PEMs). Initially, proton conductivity measurements of membranes containing different amounts of ZIF-67 (0.5, 1, 5, and 10 wt.%) were performed on the phosphoric acid-doped composite membranes between 20 and 120 °C by impedance spectroscopy (Figures S20–S26, Supporting Information) [56,57]. The proton conductivity increased with the ZIF content, reaching a maximum conductivity for the membrane containing 5 wt.% of ZIF-67. For higher contents of ZIF, i.e., 10 wt.%, a decrease in proton conductivity was observed, which may be caused by the agglomeration of ZIF particles, as observed by TEM and described in other systems. Next, a conductivity study between 0 and 200 °C (under anhydrous conditions) was carried out on the composite membranes containing the optimized amount of ZIF (5 wt.%). As can be seen in Figure 6, the conductivities increased with temperature for the three composite membranes, showing a linear temperature dependence between 20 and 100 °C and reaching a plateau around 150–160 °C, which may be attributed to the evaporation of the phosphoric acid. In all cases, the proton conductivities were higher than those measured for the pristine PBI membrane. In the case of the PBI@ZIF-8 composite membrane, values of (3.1 ± 0.2) × 10^−3^ S·cm^−1^ were reached. These are in the same range as other PEMs containing ZIF-8, but considerably higher than those of pure ZIF-8 (σ_dc_ = (4.6 × 10^−4^ S·cm^−1^ at T = 94 °C and 100% RH) [58]. The proton conductivity for the PBI@ZIF-67 membrane also increased with temperature, reaching a plateau at (4.1 ± 0.2) × 10^−2^ S·cm^−1^, which is 10-fold that observed for the PBI@ZIF-8 membrane. This enhancement can be attributed to the higher affinity of phosphonate anions to bind to Co (II) rather than to Zn (II), such as has been observed in the study of H_3_PO_4_ uptake and swelling ratios. Finally, the hybrid PBI@ZIF-mix membrane showed the highest proton conductivity with a maximum of (9.1 ± 0.2) × 10^−2^ S·cm^−1^ at 200 °C, showing a synergistically promotion of proton conductivity. The observed proton conductivities for the PBI@ZIF-67 and PBI@ZIF-mix membranes are higher than values reported for other HT-PEMFCs based on MOFs under anhydrous conditions (Tables S1–S3, Supporting Information).

From the Arrhenius plot (Figures S27–S30, Supporting Information), the calculated activation energies associated with the proton transport in the PBI@ZIF-8 and PBI@ZIF-67 were 33 ± 2 and 30 ± 2 kJ·mol^−1^, respectively. In the case of the PBI@ZIF-mix, the activation energy was 19.6 ± 1.4 kJ·mol^−1^. These values are lower than those obtained for the pure PBI membrane (E_a_ = 36 ± 2 kJ·mol^−1^), and similar to those obtained for the ZIFs doped with tetrabutylammonium hydroxide used as fillers in a polyetherimide (PEI) polymeric matrix [59], those obtained for phosphoric acid doped polybenzimidazole/imidazolium-modified silsesquioxane membranes [60], and those obtained for the SPEEK composite membranes [42], but they are higher than those for the Nafion^®^-117 membranes (E_a_ = 12 kJ·mol^−1^). Considering that the activation energy for the PBI@ZIF membranes is lower than that for pure PBI membrane, we suggest that the ZIFs clearly favor the conductivity of the membrane at moderate and high temperatures.

The proton conductivity at a molecular level in solid compounds and porous polymers can be interpreted by the Grotthuss (also known as the hopping mechanism) and vehicle mechanisms [61]. Herein, we propose that the proton conductivity in ZIF-containing PBI composite membranes could be classified mainly as the Grotthuss mechanism, although a vehicle mechanism could also be present. A vehicle mechanism can be described for the transfer of the proton from the surface of the ZIF through the next ZIF by phosphoric acid molecules as carriers. On the other hand, the Grotthuss mechanism is based on proton conductivity related to proton hopping from one proton carrier site to a neighboring one through a network of hydrogen bonds [62]. In this regard, different types of hydrogen bonds are proposed based on polybenzimidazole polymeric chains, phosphoric acid networks and imidazolate rings from the ZIF.

## 4. Conclusions

As shown, we have prepared and characterized novel PBI membranes containing ZIF-8, ZIF-67, and ZIF-mix as PEMs. The thermal degradation results indicate that these composite membranes can be used up to 250 °C. Regarding proton transport, the PBI@ZIF-67 composite membrane displayed higher proton conductivities than the PBI@ZIF-8 membrane (3.1 × 10^−3^ vs. 4.1 × 10^−2^ S·cm^−1^ at 200 °C, respectively). The observed proton conductivities increased with temperature, maintaining their conductivity even under anhydrous conditions at high temperatures. It was found that the incorporation of the ZIF-mix was more beneficial to the proton conductivity enhancement than compared to the corresponding single doping. The mixed membrane showed the highest value of 9.1 × 10^−2^ S·cm^−1^ at 200 °C. This exceeded the conductivities reported for other HT-PEMFCs based on MOFs under anhydrous conditions. These results pave the way for further studies oriented towards the quest for novel high-performance MOF-based proton conducting polymeric membranes that operate in the temperature window of 120–180 °C with the benefits of working at moderate and high temperatures. This would make them suitable for use in membrane electrode assemblies for automotive applications.

## Figures and Tables

**Table 1:** Mechanical properties of polybenzimidazole (PBI) and PBI@ZIF composite membranes (5 wt.% of ZIF) measured at room temperature.

Membrane	Young’s Modulus (GPa)	Tensile Strength (MPa)	Elongation at Break (%)	Toughness (MJ·m^−3^)
PBI dry	2.52 ± 0.17	174 ± 4	2.0 ± 0.1	0.5 ± 0.1
PBI (75% RH)	1.22 ± 0.12	81 ± 3	242 ± 6	96 ± 2
PBI@ZIF-8	1.53 ± 0.08	79 ± 2	79 ± 5	50 ± 8
PBI@ZIF-67	1.44 ± 0.10	77 ± 3	26 ± 6	13 ± 4
PBI@ZIF-mix	1.61 ± 0.15	87 ± 2	180 ± 17	119 ± 3

## Supplementary Materials

The following supporting information including a detailed description of experimental procedures, additional tables and supporting figures is available online at http://www.mdpi.com/2079-4991/8/10/775/s1, Table S1: Comparison of proton conductivities of different membranes containing ZIFs; Table S2: Comparison of proton conductivities of Nafion^®^-based membranes containing MOFs; Table S3: Comparison of proton conductivities of SPEEK-based membranes containing MOFs; Table S4: Textural properties of ZIF materials obtained by nitrogen adsorption isotherms; Table S5: Acid leaching degree for all the membranes; Figure S1: Photograph of synthesized ZIF-8 and ZIF-67; Figure S2: Photograph of PBI composite membranes containing 5 wt.% of ZIF; Figure S3: (a) XRD patterns of simulated ZIF-8, ZIF-8 as synthesized and PBI@ZIF-8 membrane; (b) XRD patterns of simulated ZIF-67, ZIF-67 as synthesized and PBI@ZIF-7 membrane; Figures S4-S6: EDX of the PBI@ZIF composite membranes with 5 wt.% loading of ZIF; Figures S7-S11: FT-IR spectra of ZIFs and PBI@ZIF composite membranes with 5 wt. % loading of ZIF; Figures S12–S15: Thermogravimetric analysis of different samples; Figure S16: Stress-strain curves of PBI (dry and 75% RH) and composite PBI@ZIF membranes; Figure S17: H_3_PO_4_ uptake and swelling ratios of PBI and PBI composite membranes containing different contents (wt.%) of ZIF-67; Figure S18: Thermogravimetric analysis of PA-doped PBI membrane and PA-doped PBI composite membranes; Figure S19: Comparison of PBI proton conductivity before (blue spots) and after (red spots) phosphoric acid doping; Figure S20: Comparison of proton conductivity under wet conditions for composite phosphoric acid PA-doped PBI membranes containing ZIF-67 at different content (0.5, 1, 5 and 10 wt.%); Figures S21–S26: Conductivity (S·cm^−1^) vs. frequency (Hz) for PBI@ZIF composite membranes with 5 wt.% of ZIF at different temperatures; Figure S27: Arrhenius plots for pure PBI membrane and PBI@ZIF composite membranes.
