# Supplementary material for: Phosphoric Acid Doped Polybenzimidazole (PBI)/Zeolitic Imidazolate Framework Composite Membranes with Significantly Enhanced Proton Conductivity under Low Humidity Conditions
Source: Nanomaterials (Basel). 2018 Sep 29;8(10):775. doi: 10.3390/nano8100775 (PMC6215102; doi:10.3390/nano8100775)
Supplement: Supplementary file 1 [file nanomaterials-08-00775-s001.pdf]

Supporting Information

Phosphoric Acid Doped Polybenzimidazole (PBI)/Zeolitic Imidazolate Framework Composite Membranes with Significantly Enhanced Proton Conductivity under Low Humidity Conditions

Jorge Escorihuela [1], Óscar Sahuquillo [2], Abel García-Bernabé [1], Enrique Giménez [2] and Vicente Compañ [1,]*

[1] Escuela Técnica Superior de Ingenieros Industriales – Departamento de Termodinámica Aplicada, Universitat Politècnica de València, Camino de Vera s/n, 46020 Valencia, Spain; escorihu@uji.es (J.E.); agarciab@ter.upv.es (A.G.-B.)
[2] Instituto de Tecnología de Materiales, Universitat Politècnica de València, Camino de Vera s/n, 46020 Valencia, Spain; ossana@upvnet.upv.es (O.S.); enrique.gimenez@mcm.upv.es (E.G.)
* Correspondence: vicommo@ter.upv.es; Tel.: +34-96-387-9328

1. Supplementary Tables

Table S1. Comparison of proton conductivities of different membranes containing zeolitic imidazolate framework (ZIFs).

Polymer	MOF	Additive	Conditions	Conductivity ($S \cdot cm^{-1}$)	Ref.
polyetherimide (PEI)	ZIF-8	TBA	90 °C, anhydrous	1.2×10^{-4}	1
PEI	ZIF-67	TBA	90 °C, anhydrous	0.8×10^{-4}	1
PEI	ZIF-mix	TBA	90 °C, anhydrous	0.8×10^{-5}	1
poly(vinylalcohol) (PVA)	ZIF-8	PAMS	80 °C, 100 % RH	0.134	2
PVPA	ZIF-8	-----	140 °C, anhydrous	3.2×10^{-3}	3
Nafion	ZIF-8	GO	120 °C, 40 % RH	0.28	4
sulfonated poly(ether ether ketone) (SPEEK)	ZIF-8	-----	120 °C, 40 % RH	0.025	5
SPEEK	ZIF-8	CNTs	120 °C, 430 % RH	0.05	5
PBI	ZIF-8	H_3PO4	180 °C, anhydrous	3.1×10^{-3}	This work
PBI	ZIF-67	H_3PO4	180 °C, anhydrous	0.042	This work
PBI	ZIF-mix	H_3PO4	180 °C, anhydrous	0.092	This work

Table S2. Comparison of proton conductivities of Nafion-based membranes containing MOFs.

MOF	Additive	Conditions	Conductivity ($S \cdot cm^{-1}$)	Ref.
Nafion	ZIF-8, GO	120 °C, 40 % RH	0.28	4
MIL-101	Phytic acid	100 °C, 100% RH	0.228	6
CPO-27-Mg	-----	50 °C, 100% RH	0.011	7
MIL-53	-----	50 °C, 100% RH	9.8×10^{-3}	7
HKUST-1	H_3PO_4	25 °C, 100% RH	0.018	8
MOF-808- SO_3H	-----	80 °C, 35% RH	2.98×10^{-3}	9
UiO-66-NH_2	graphene oxide (GO)	120 °C, anhydrous	3.4×10^{-3}	10
2:1 UiO-66-NH_2:UiO-66- SO3H	-----	90 °C, 95% RH	0.256	11
UiO-66-SO_3H	-----	80 °C, 95% RH	0.17	12

Table S3. Comparison of proton conductivities of SPEEK-based membranes containing MOFs.

MOF	Additive	Conditions	Conductivity ($S\cdot cm^{-1}$)	Ref.
MIL-101	Phosphotungstic acid	65 °C, 100% RH	0.272	13
MIL-101-SO_3H	-----	70 °C, 100% RH	0.306	14
UiO-66- SO_3H	GO	70 °C, 95% RH	0.173	5

Table S4. Textural properties of ZIF materials obtained by nitrogen adsorption isotherms.

Sample	BET surface area ($m^2\cdot g^{-1}$)	Langmuir surface area ($m^2\cdot g^{-1}$)	Micropore volume ($cm^3\cdot g^{-1}$)
ZIF-8	1150.73	1540.83	0.572
ZIF-67	1378.77	1816.52	0.634
ZIF-mix	1179.40	1546.93	0.577

Table S5. Acid leaching degree for all the membranes.

Membrane	Acid leaching degree (%)
PBI	36
PBI@ZIF-8 5 wt.%	28
PBI@ZIF-67 5 wt.%	29
PBI@ZIF-mix 5 wt.%	30

2. Supplementary Figures.

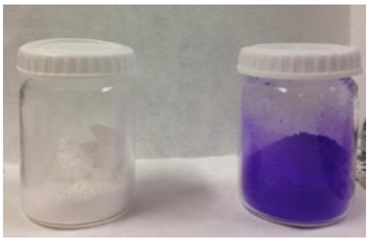

ZIF-8 **ZIF-67**

Figure S1. Photograph of synthesized ZIF-8 and ZIF-67.

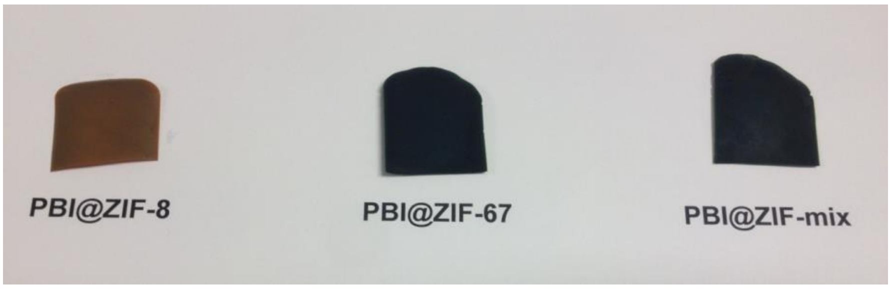

Figure S2. Photograph of PBI composite membranes containing 5 wt.% of ZIF-8 (PBI@ZIF-8), ZIF-67 (PBI@ZIF-67) and a binary mixture of ZIF-8 and ZIF-67 (PBI@ZIF-mix).

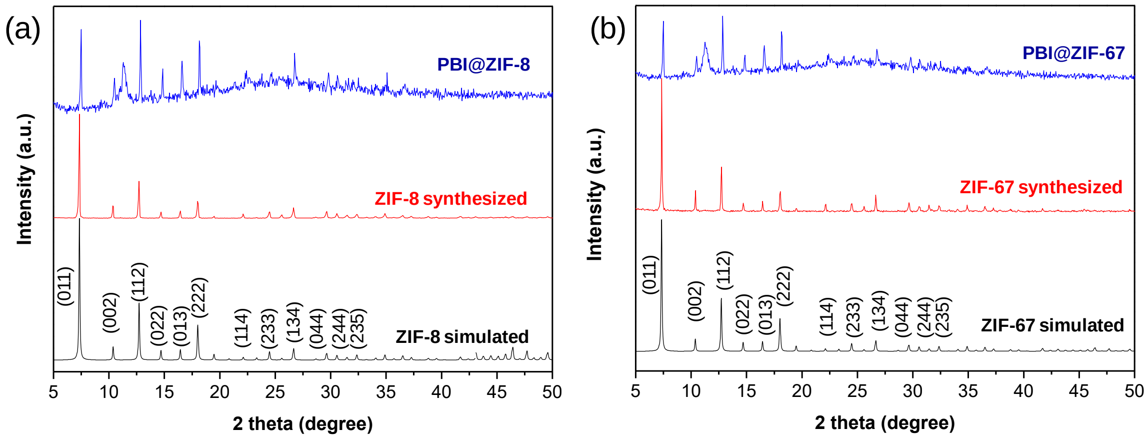

Figure S3. (a) XRD patterns of simulated ZIF-8 (black), ZIF-8 as synthesized (red) and PBI@ZIF-8 membrane (blue); (b) XRD patterns of simulated ZIF-67 (black), ZIF-67 as synthesized (red) and PBI@ZIF-7 membrane (blue).

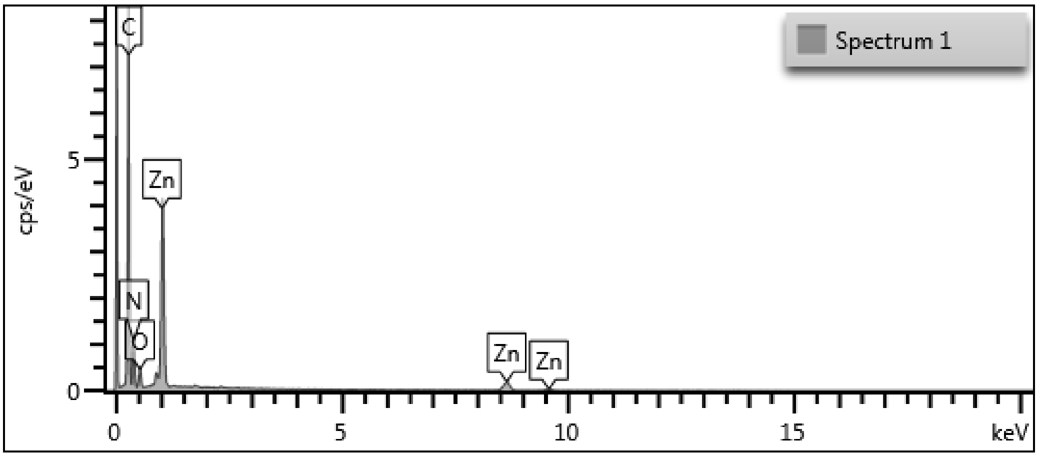

Figure S4. EDX of the PBI@ZIF-8 membrane with 5 wt. % loading of ZIF-8.

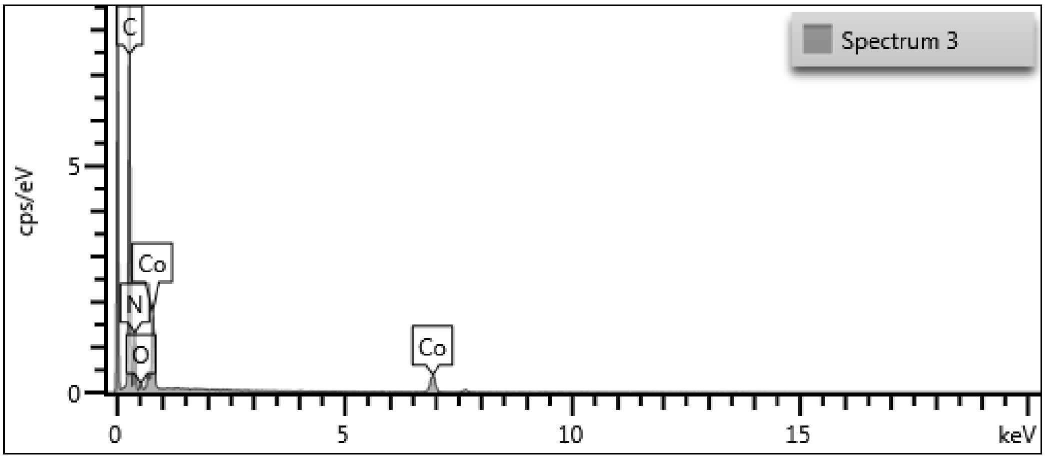

Figure S5. EDX of the PBI@ZIF-67 membrane with 5 wt.% loading of ZIF-67.

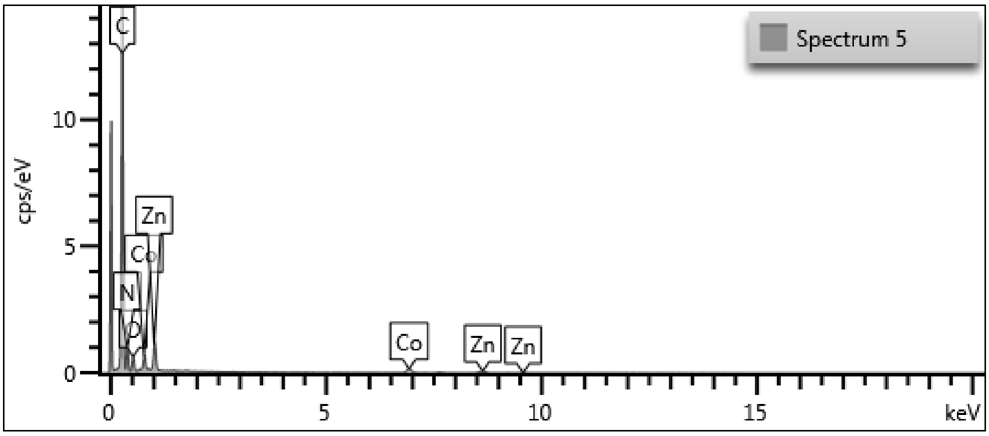

Figure S6. EDX of the PBI@ZIF-mix membrane with 5 wt.% loading of ZIF-mix.

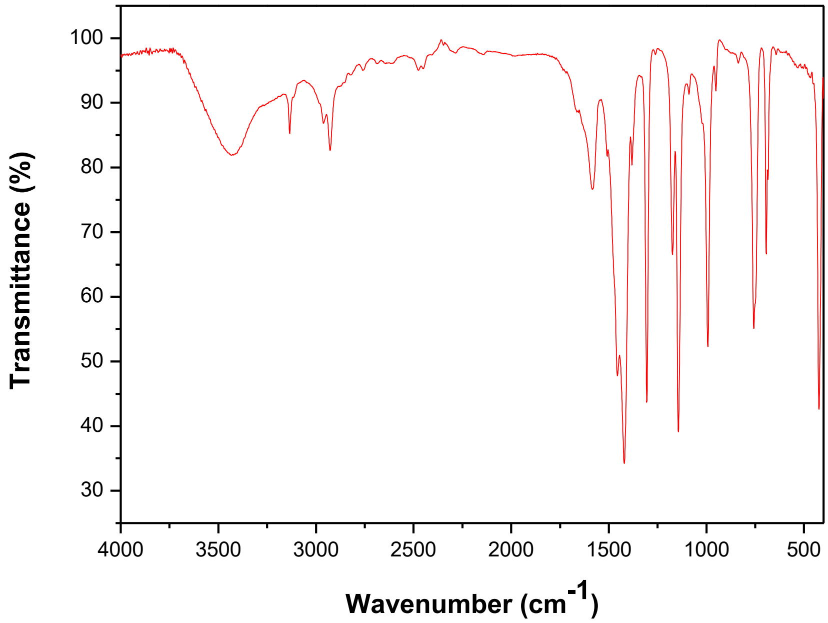

Figure S7. FT-IR spectra of ZIF-8.

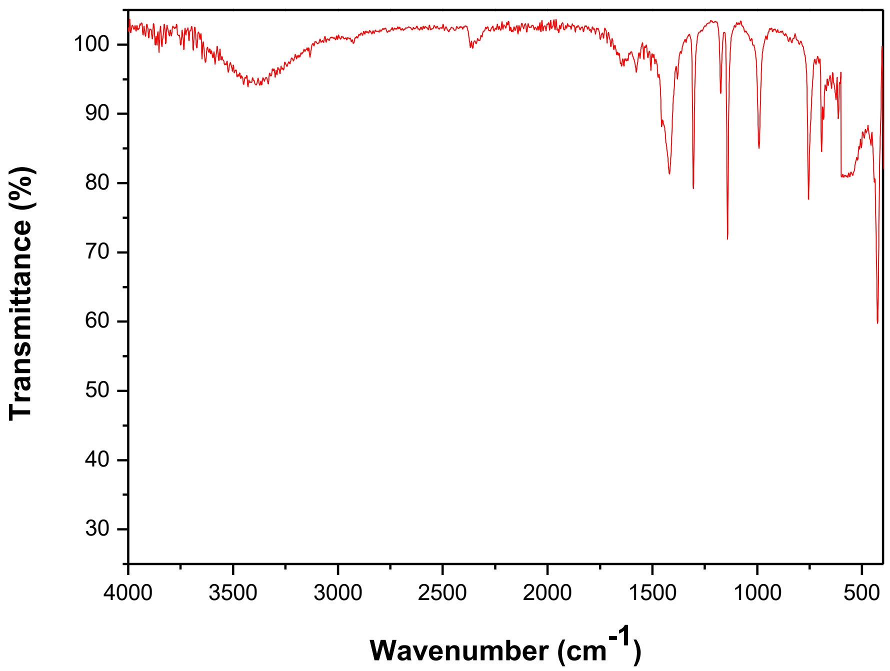

Figure S8. FT-IR spectra of ZIF-67.

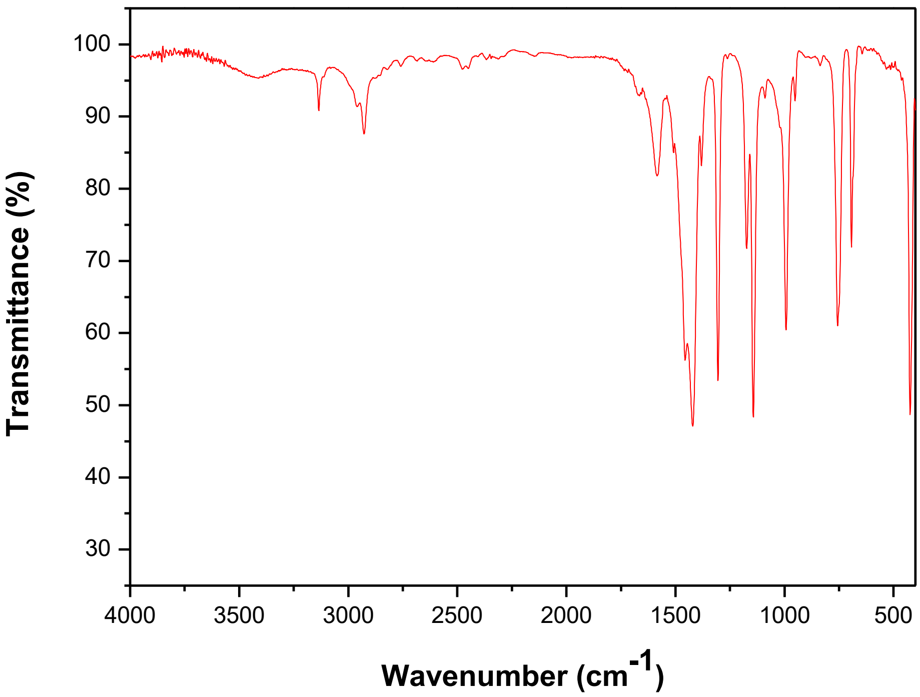

Figure S9. FT-IR spectra of ZIF-mix.

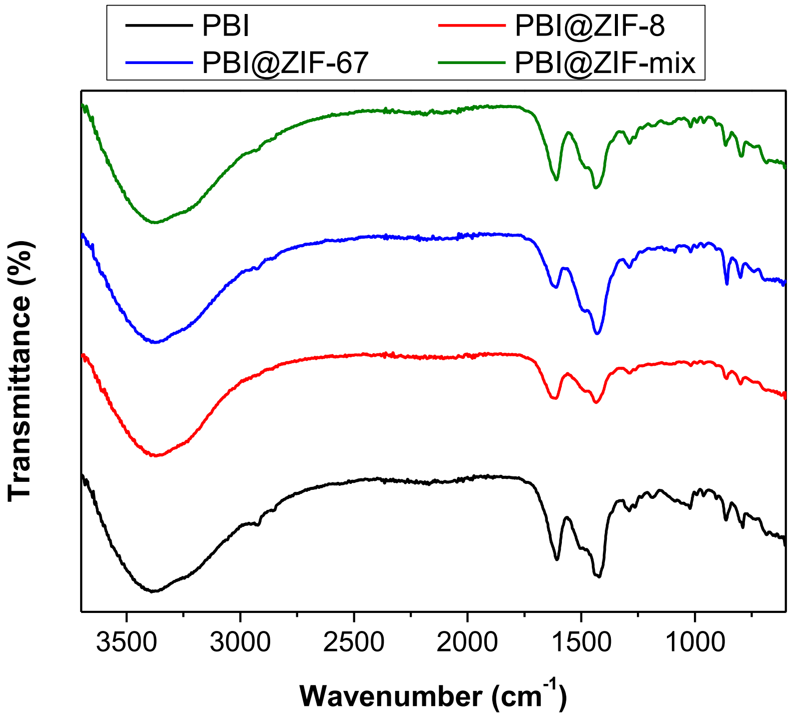

Figure S10. FT-IR spectra of composite membranes PBI@ZIF-8, PBI@ZIF-67 and PBI@ZIF-mix. All membranes contain the ZIF compound at 5 wt.%.

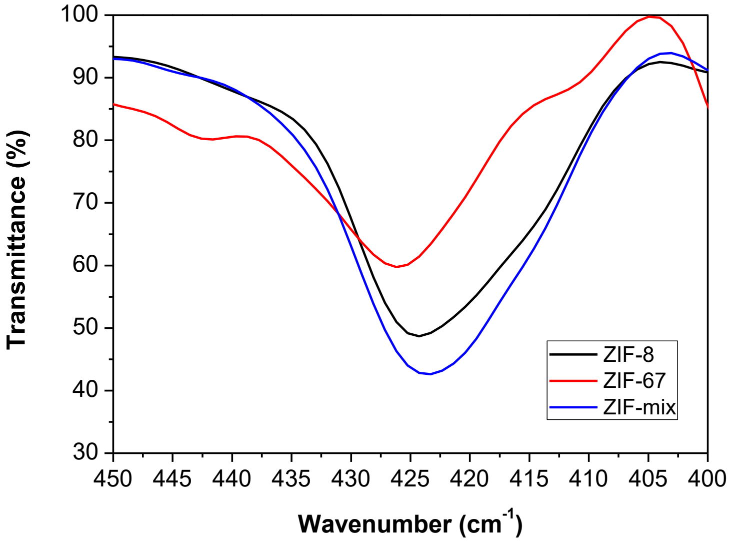

Figure S11. Comparison of 450-400 cm^{-1} region for FT-IR spectra of composite membranes PBI@ZIF-8, PBI@ZIF-67 and PBI@ZIF-mix. All membranes contain the ZIF compound at 5 wt.%.

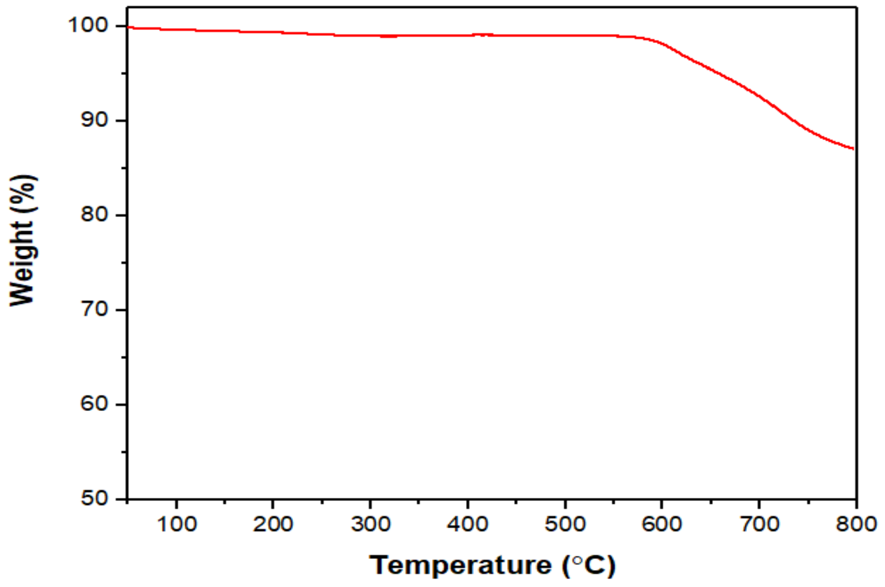

Figure S12. Thermogravimetric analysis of pristine PBI.

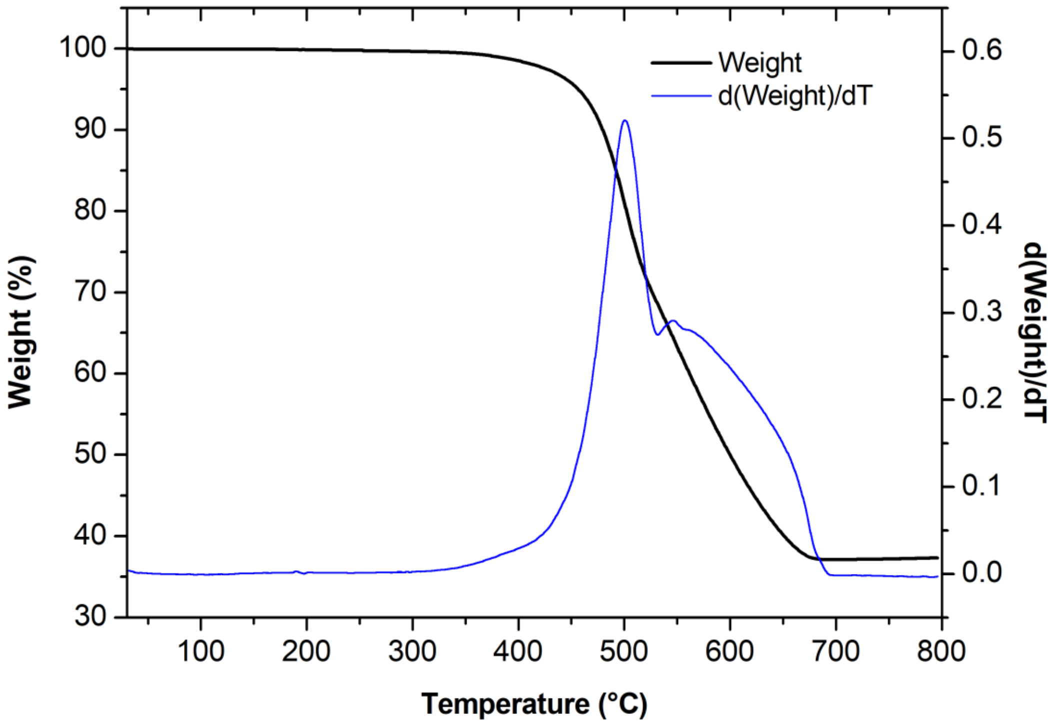

Figure S13. Thermogravimetric analysis of ZIF-8 compound.

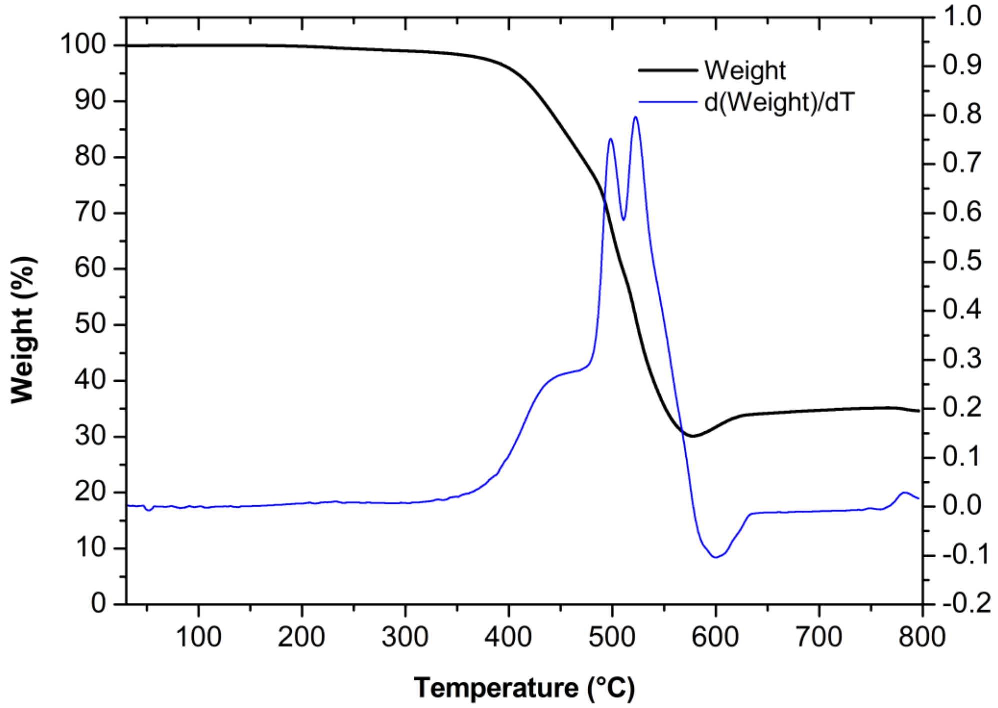

Figure S14. Thermogravimetric analysis of ZIF-67 compound.

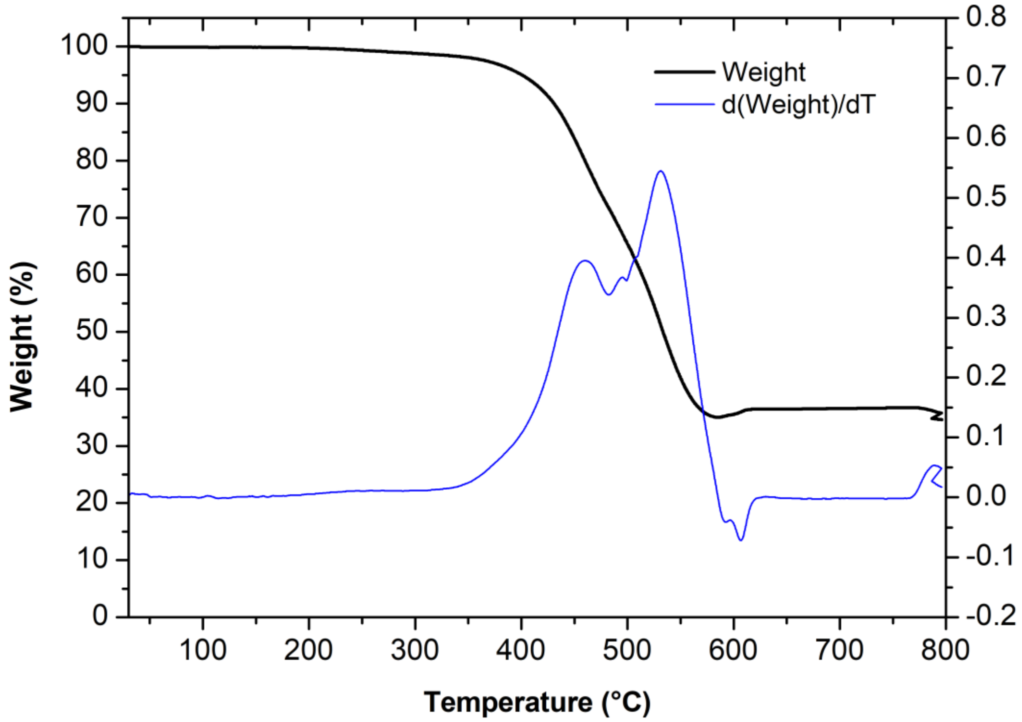

Figure S15. Thermogravimetric analysis of ZIF-mix.

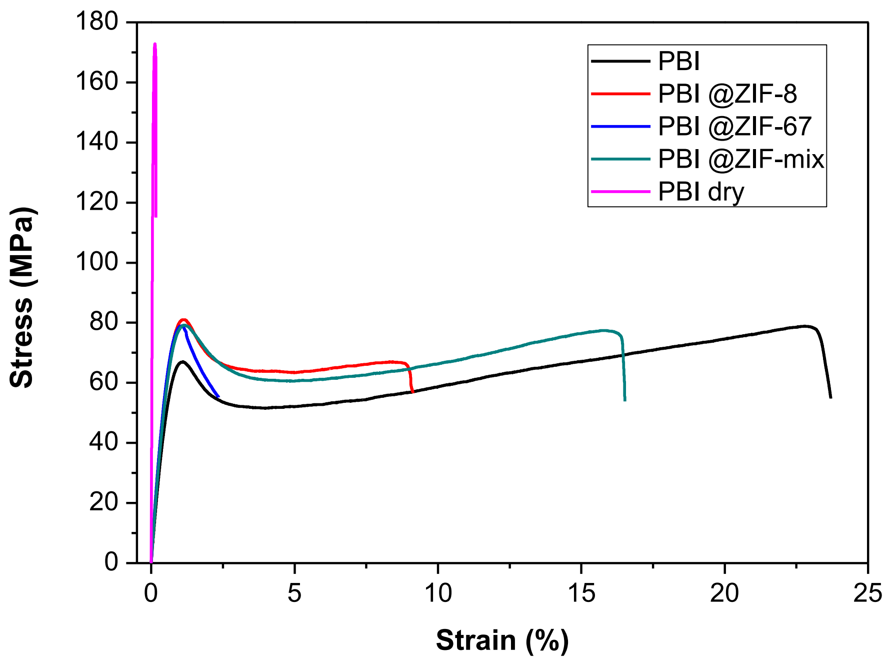

Figure S16. Stress-strain curves of PBI (dry and 75% RH) and composite membranes PBI@ZIF-8, PBI@ZIF-67 and PBI@ZIF-mix. All membranes contain the ZIF compound at 5 wt.%.

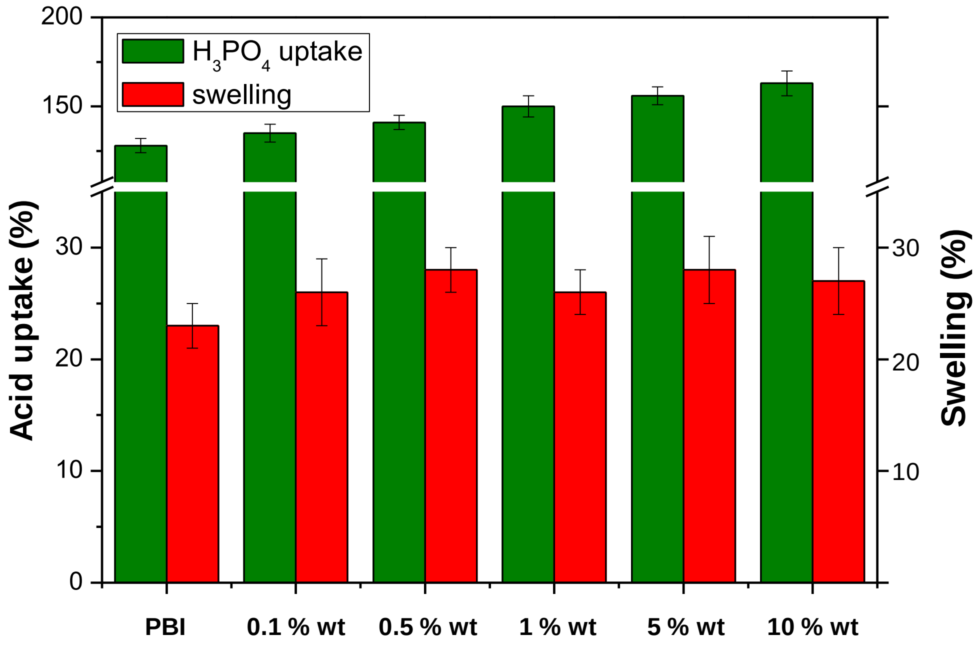

Figure S17. H_3PO_4 uptake and swelling ratios of PBI and PBI composite membranes containing different contents (wt.%) of ZIF-67.

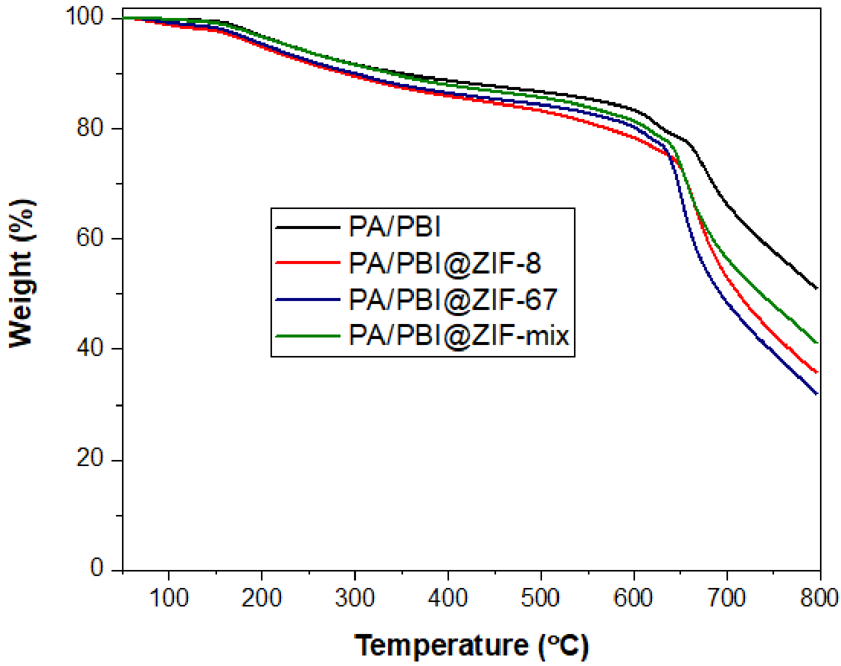

Figure S18. Thermogravimetric analysis of phosphoric acid doped PBI membrane and phosphoric acid doped PBI composite membranes containing 5 wt.% of ZIF-8, ZIF-67 and ZIF-mix. Inset: Zoom at the 180–600 °C region.

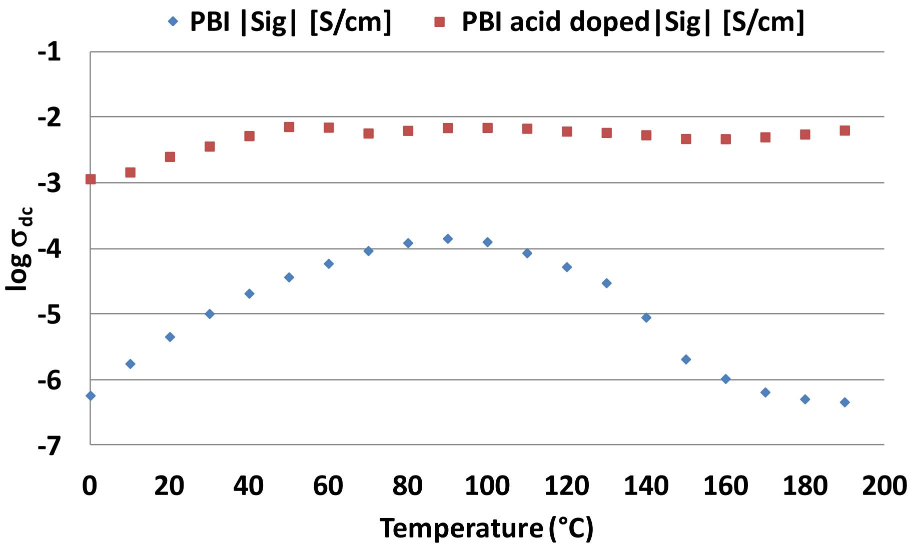

Figure S19. Comparison of PBI proton conductivity before (blue spots) and after (red spots) phosphoric acid doping.

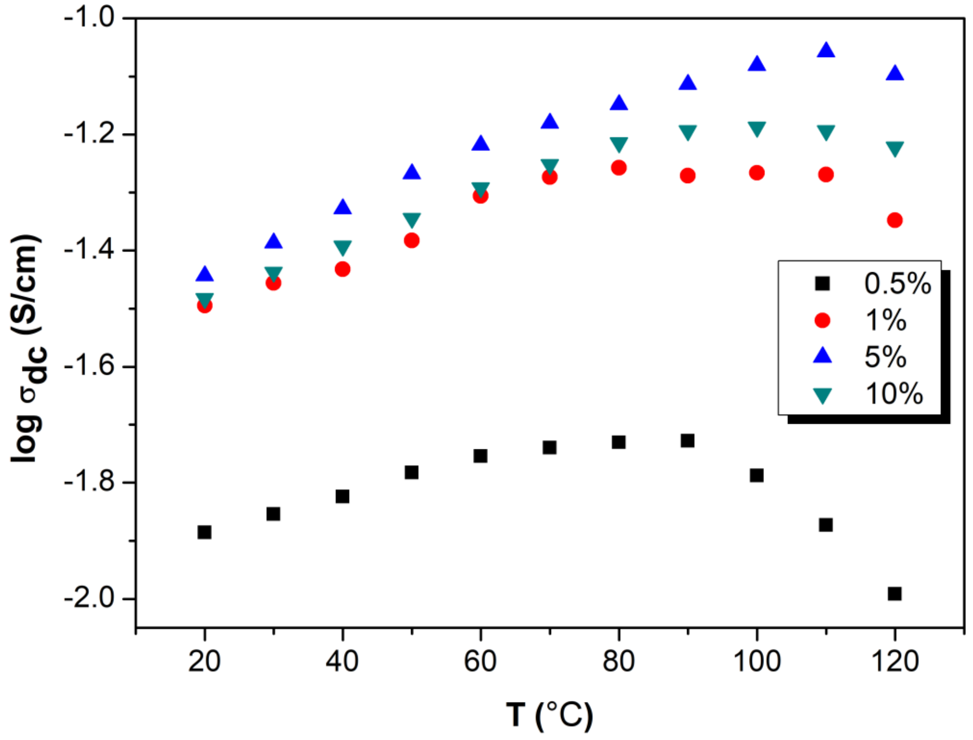

Figure S20. Comparison of proton conductivity under wet conditions for composite phosphoric acid doped PBI membranes containing ZIF-67 at different content (0.5, 1, 5 and 10 wt.%).

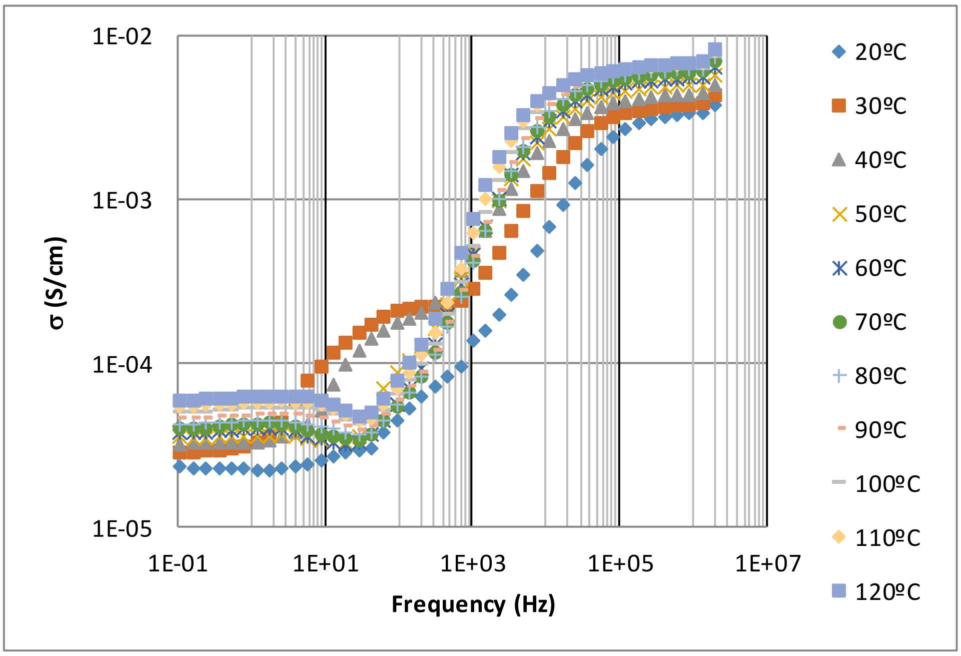

Figure S21. Conductivity (S/cm) *vs*. Frequency (Hz) for PBI@ZIF-8 with 5 wt.% content of ZIF-8 at different temperatures (20−120 °C) under wet conditions.

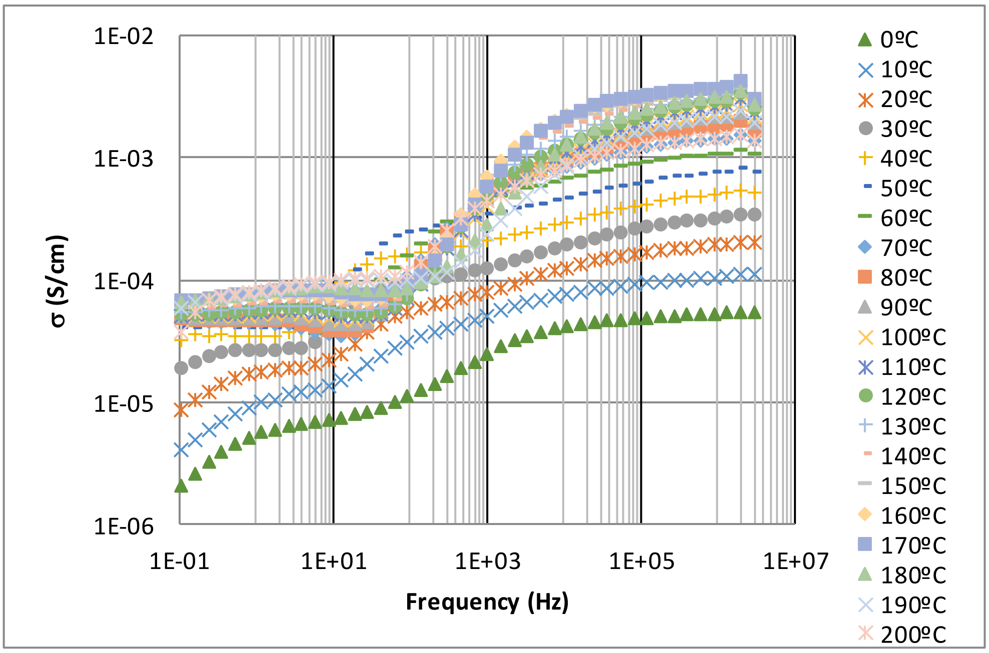

Figure S22. Conductivity (S/cm) *vs.* Frequency (Hz) for PBI@ZIF-8 with 5 wt.% content of ZIF-8 at different temperatures (0−200 °C) under anhydrous conditions.

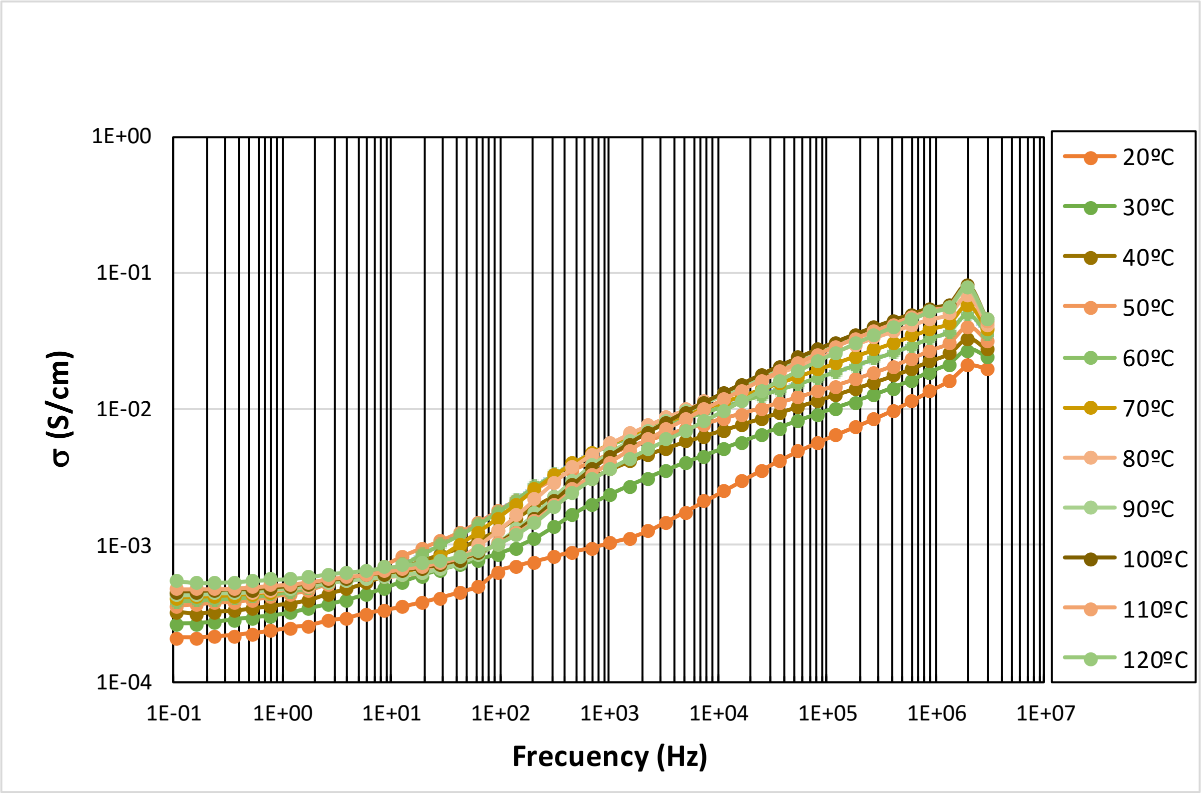

Figure S23. Conductivity (S/cm) *vs.* Frequency (Hz) for PBI@ZIF-67 with 5 wt.% content of ZIF-67 at different temperatures (20−120 °C) under wet conditions.

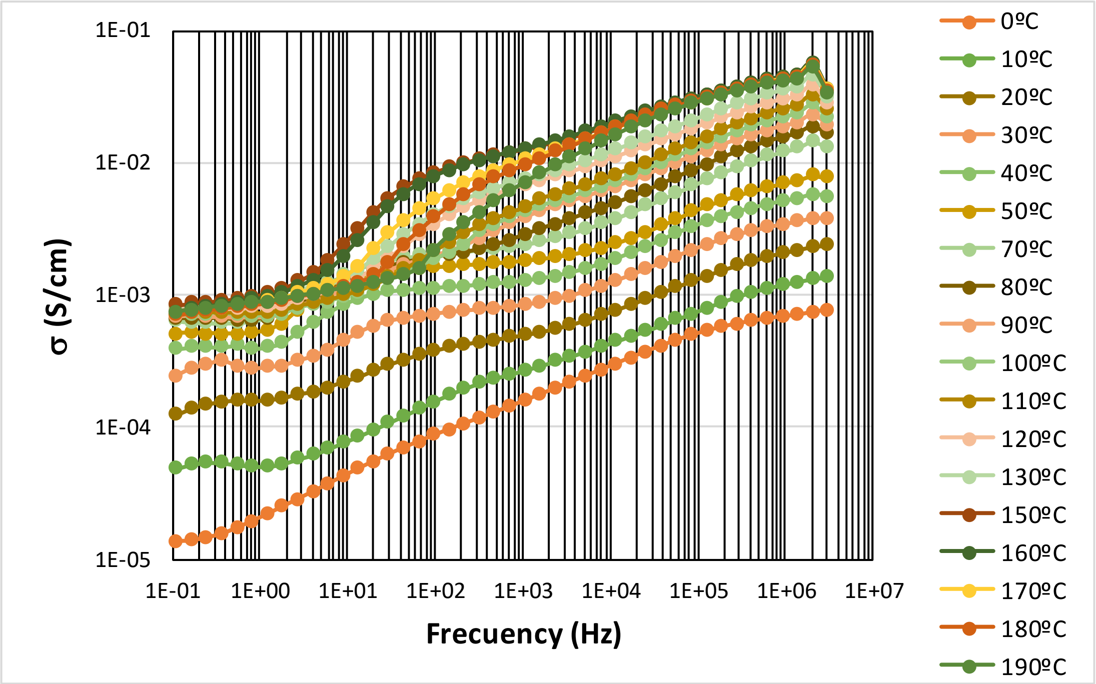

Figure S24. Conductivity (S/cm) *vs*. Frequency (Hz) for PBI@ZIF-67 with 5 wt. % content of ZIF-67 at different temperatures (0−200 °C) under anhydrous conditions.

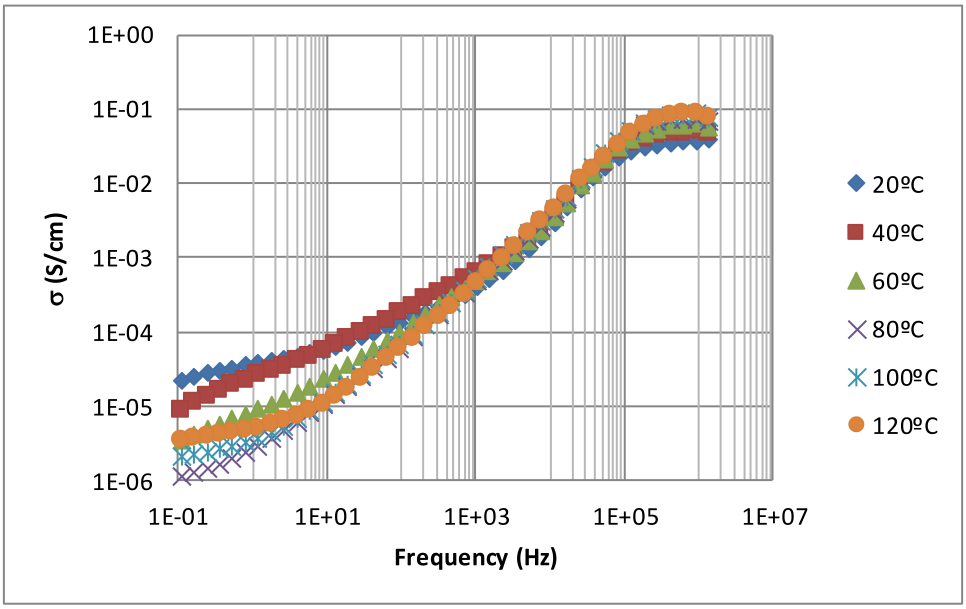

Figure S25. Conductivity (S/cm) *vs*. Frequency (Hz) for PBI@ZIF-mix with 5 wt.% content of ZIF-mix at different temperatures (20−120 °C) under wet conditions.

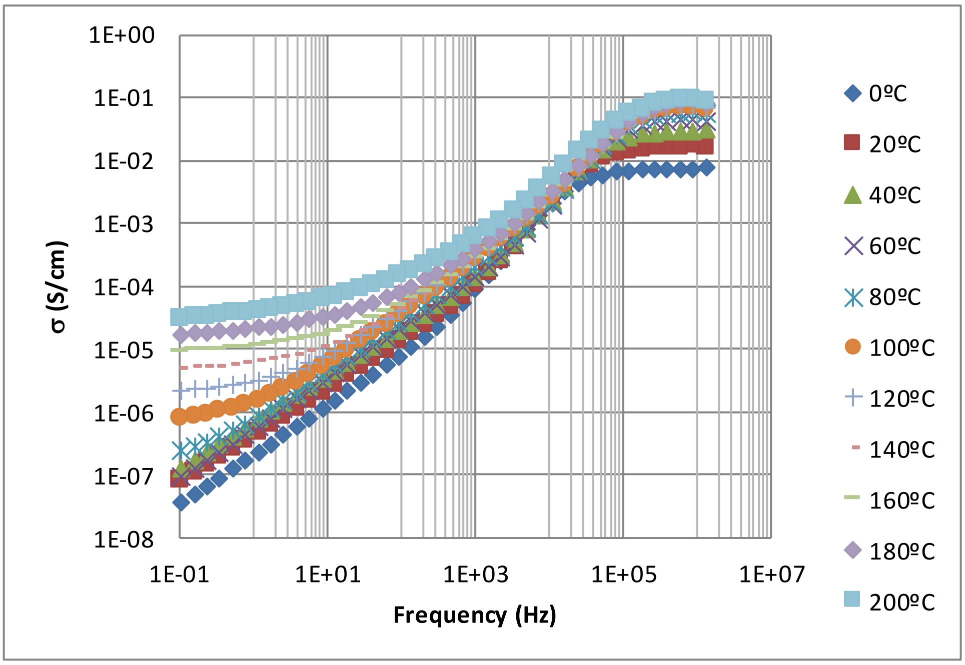

Figure S26. Conductivity (S/cm) *vs*. Frequency (Hz) for PBI@ZIF-mix with 5 wt.% content of ZIF-mix at different temperatures (0−200 °C) under anhydrous conditions.

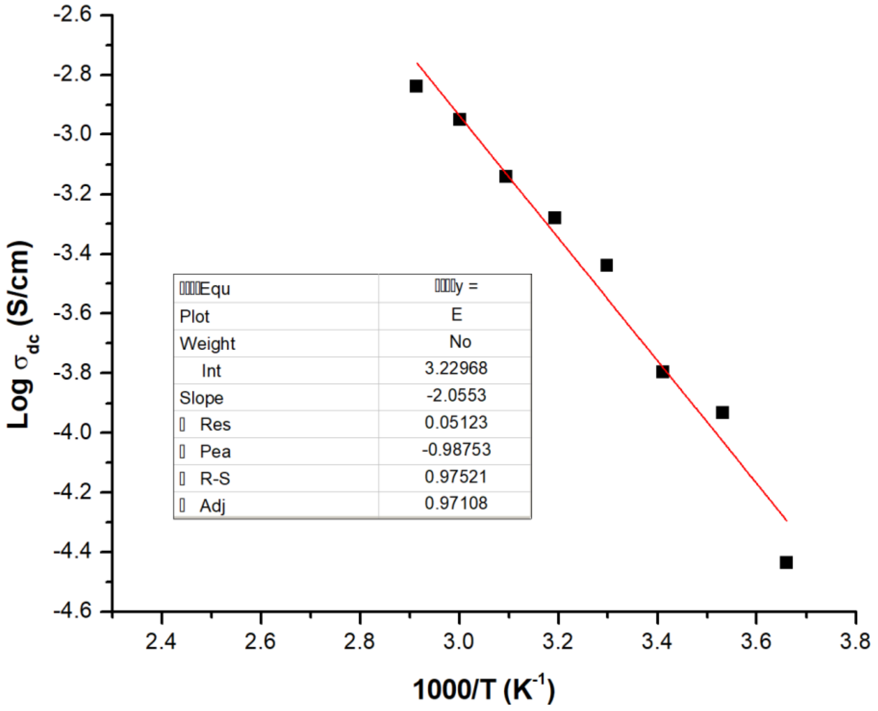

Figure S27. Arrhenius plot for pure PBI membrane.

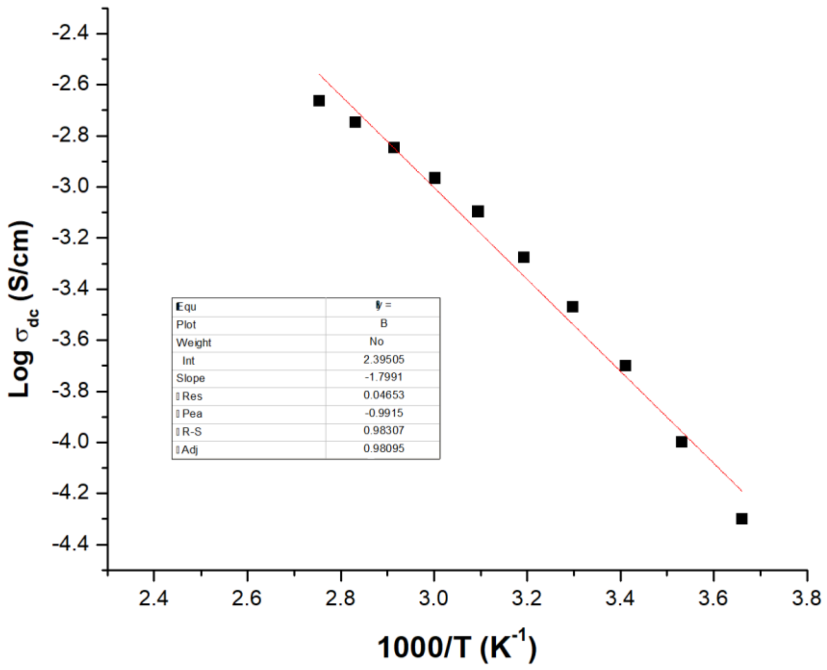

Figure S28. Arrhenius plot for PBI@ZIF-8 membrane with 5 wt.% content of ZIF-8.

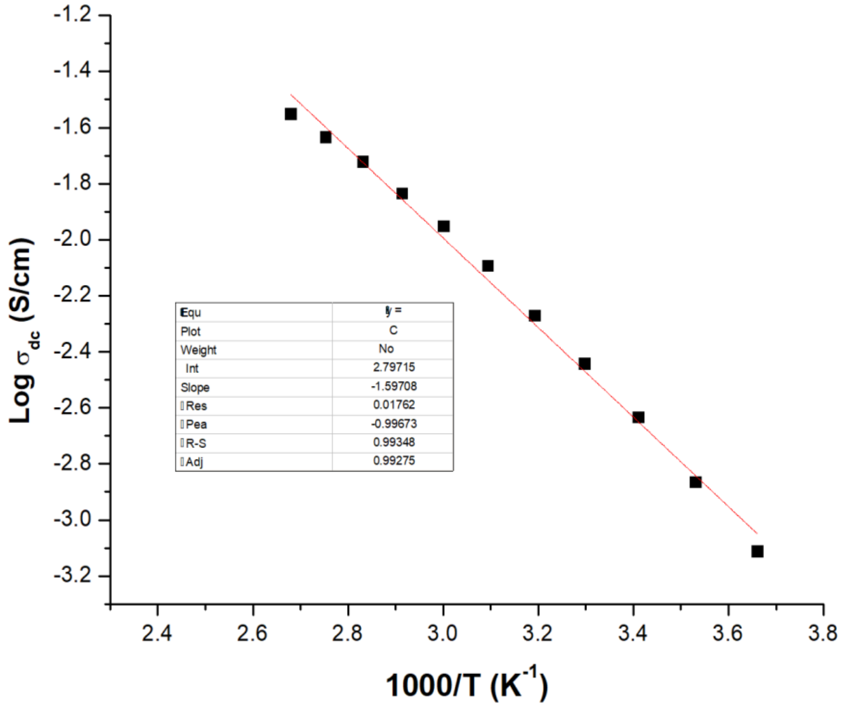

Figure S29. Arrhenius plot for PBI@ZIF-67 membrane with 5 wt.% content of ZIF-67.

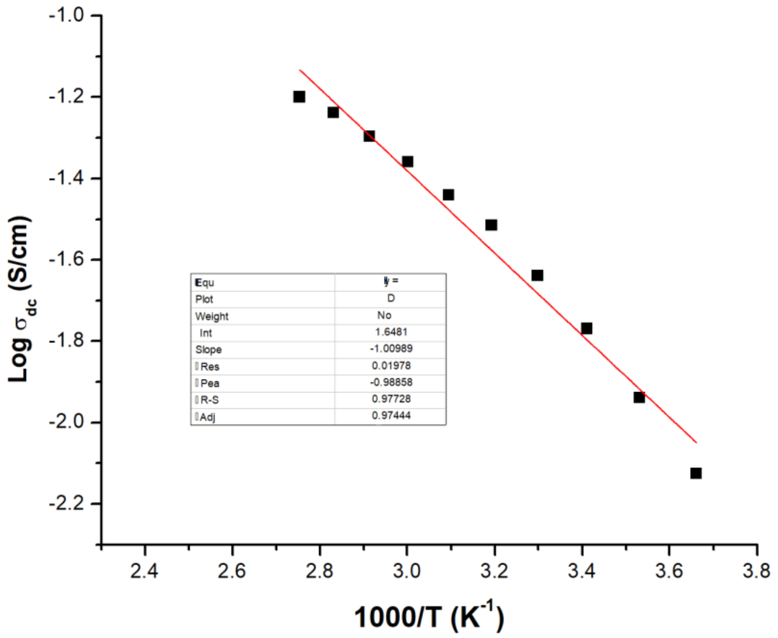

Figure S30. Arrhenius plot for PBI@ZIF-mix membrane with 5 wt.% content of ZIF-mix.

References

1. Vega, J.; Andrio, A.; Lemus, A.A.; del Castillo, L.F.; Compañ, V. Conductivity study of zeolitic imidazolate frameworks, tetrabutylammonium hydroxide doped with zeolitic imidazolate frameworks, and mixed matrix membranes of polyetherimide/tetrabutylammonium hydroxide doped with zeolitic imidazolate frameworks for proton conducting applications. *Electrochim. Acta* **2017**, *258*, 153–166.
2. Erkartal, M.; Usta, H.; Citir, M.; Sen, U. Proton conducting poly(vinyl alcohol) (PVA)/ poly(2-acrylamido-2-methylpropane sulfonic acid) (PAMPS)/ zeolitic imidazolate framework (ZIF) ternary composite membrane. *J. Membr. Sci*. **2016**, *499*, 156–163.
3. Sen, U.; Erkartal, M.; Kung, C.-W.; Ramani, V.; Hupp, J.T.; Farha, O.K. Proton conducting self-assembled metal–organic framework/polyelectrolyte hollow hybrid nanostructures. *ACS Appl. Mater. Interfaces* **2016**, *8*, 23015–23021.
4. Yang, L.; Tang, B.; Wu, P. Metal–organic framework–graphene oxide composites: a facile method to highly improve the proton conductivity of PEMs operated under low humidity. *J. Mater. Chem. A* **2015**, *3*, 15838–15842.
5. Sun, H.; Tang, B.; Wu, P. Two-Dimensional Zeolitic imidazolate framework/carbon nanotube hybrid networks modified proton exchange membranes for improving transport properties. *ACS Appl. Mater. Interfaces* **2017**, *9*, 35075–35085.
6. Sun, H.; Tang, B.; Wu, P. Rational design of S-UiO-66@GO hybrid nanosheets for proton exchange membranes with significantly enhanced transport performance. *ACS Appl. Mater. Interfaces* **2017**, *9*, 26077–26087.
7. Li, Z.; He, G.; Zhang, B.; Cao, Y.; Wu, H.; Jiang, Z.; Tiantian, Z. Enhanced proton conductivity of Nafion hybrid membrane under different humidities by incorporating metal–organic frameworks with high phytic acid loading. *ACS Appl. Mater. Interfaces* **2014**, *6*, 9799–9807.
8. Kim, H.J.; Talukdar, K.; Choi, S.J. Tuning of Nafion® by HKUST-1 as coordination network to enhance proton conductivity for fuel cell applications *J. Nanopart. Res*. **2016**, *18*, 47.
9. Patel, H.A.; Mansor, N.; Gadipelli, S.; Brett, D.J.L.; Guo, Z. Superacidity in Nafion/MOF hybrid membranes retains water at low humidity to enhance proton conduction for fuel cells. *ACS Appl. Mater. Interfaces* **2016**, *8*, 30687–30691.
10. Rao, Z.; Feng, K.; Tang, B.; Wu, P. Construction of well interconnected metal-organic framework structure for effectively promoting proton conductivity of proton exchange membrane. *J. Membr. Sci*. **2017**, *533*, 160–170.
11. Rao, Z.; Tang, B.; Wu, P. Proton conductivity of proton exchange membrane synergistically promoted by different functionalized metal–organic frameworks. *ACS Appl. Mater. Interfaces* **2017**, *9*, 22597–22603.
12. Donnadio, A.; Narducci, R.; Casciola, M.; Marmottini, F.; D'Amato, R.; Jazestani, M.; Chiniforoshan, H.; Costantino, F. Mixed membrane matrices based on Nafion/UiO-66/SO_3H-UiO-66 nano-MOFs: revealing the effect of crystal size, sulfonation, and filler loading on the mechanical and conductivity properties. *ACS Appl. Mater. Interfaces* **2017**, *9*, 42239–42246.
13. Zhang, B.; Cao, Y.; Li, Z.; Wu, H.; Yin, Y.; Cao, L.; He, X.; Jiang, Z. Proton exchange nanohybrid membranes with high phosphotungstic acid loading within metal-organic frameworks for PEMFC applications. *Electrochim. Acta* **2017**, *240*, 186–194.
14. Li, Z.; He, G.; Zhao, Y.; Cao, Y.; Wu, H.; Li, Y.; Jiang, Z. Enhanced proton conductivity of proton exchange membranes by incorporating sulfonated metal-organic frameworks. *J. Power Sources* **2014**, *262*, 372–379.
